# H‐Bonding‐Induced Repulsion Between Small Groups Toward a Highly Puckered Benzene

**DOI:** 10.1002/advs.78352

**Published:** 2026-10-18

**Authors:** Chaofeng Zhao, Qi Sun, Ning Ding, Xiaoting Ren, Jinxuan He, Shenghua Li, Siping Pang

**Affiliations:** ^1^ School of Materials Science and Engineering Beijing Institute of Technology Beijing China; ^2^ Department of Synthesis Hubei Institute of Aerospace Chemical Technology Xiangyang China

**Keywords:** benzene ring puckering, energetic materials, hydrogen bonding, steric repulsion

## Abstract

Benzene ring puckering generally arises from the strong repulsion between bulky substituents, while in this study, we present that benzene ring puckering can be achieved by repulsion between small substituents (nitro and methyl groups) facilitated by hydrogen‐bonding attraction, as exemplified in 1,3,5‐trinitro‐2,4,6‐trimethylaminobenzene. The role of intramolecular hydrogen bonding and steric repulsion is further investigated through comparison with four structurally related compounds differing in hydrogen‐bond donor availability and substituent size.

## Graphical abstract

A benzene derivative bearing methylamino and nitro substituents adopts a boat‐shaped ring conformation. Comparison with four related compounds supports a role for intramolecular hydrogen bonding in constraining substituent orientations and promoting local steric crowding associated with ring puckering.

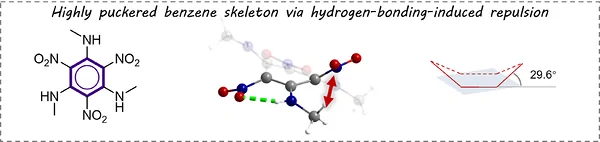

## 1 Introduction

Since the discovery of benzene by Faraday two centuries ago, its functionalization has experienced rapid development [1, 2, 3]. Countless benzene derivatives have been synthesized and reported; their rich structural features and diverse physicochemical properties have established their irreplaceable status in chemistry and materials science [4, 5, 6]. Benzene rings generally adopt a planar geometry [7, 8]. Early theoretical studies examined the electronic origins of benzene's D6h symmetry and the tendency of its π‐electron system toward symmetry‐lowering distortions, including bond‐length alternation [9, 10]. These studies provide a broader electronic perspective on benzene deformation. In certain derivatives, the ring also exhibits out‐of‐plane deformation, specifically termed ring puckering [11, 12, 13, 14]. For example, Hashimoto et al. reported a boat‐shaped puckered benzene ring in 3b,15b‐bis(thiophospha)‐9,10,21,22‐tetramethyldibenzo[de, op] diphenaleno[3,2,1‐hi:3',2',1'‐st] pentacene with a twist angle of 23.0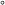 [11], while Sakurai et al. observed a chair‐shaped benzene ring in hexakis(trimethylsilyl)benzene with twist angles of 6.6° and 10.2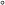 [12] (Figure 1A).

**FIGURE 1:**
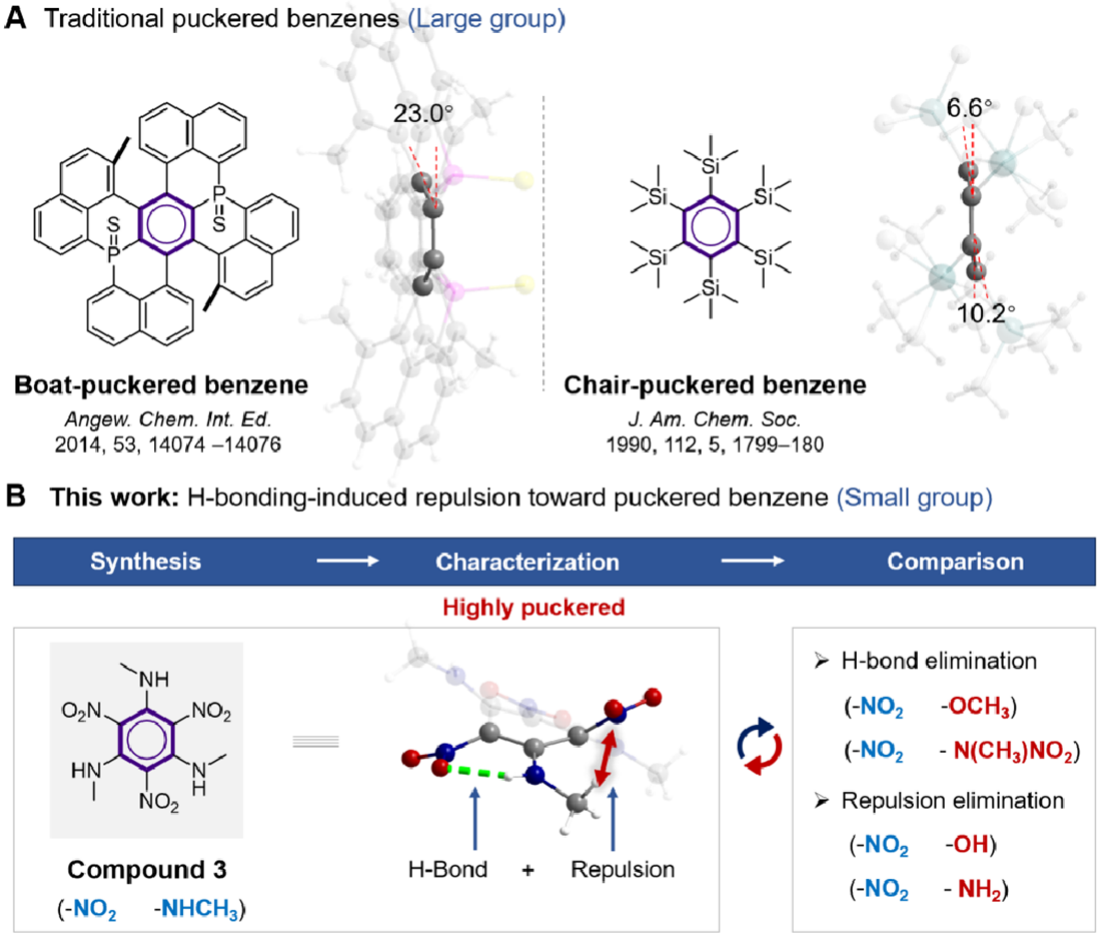
Research background of puckered benzenes and the conceptual design of this work. (A) Representative puckered benzene derivatives driven by severe steric hindrance, exemplified by boat‐ and chair‐shaped conformations with their respective twist angles. (B) This work: proposed relationship between intramolecular hydrogen bonding, local steric crowding, and benzene ring puckering.

Steric crowding between bulky substituents is an established route to benzene ring puckering [11, 15]. In contrast, many benzene derivatives bearing smaller substituents retain nearly planar rings [16, 17, 18, 19, 20], as exemplified by 1,3,5‐triamino‐2,4,6‐trinitrobenzene (TATB) [21, 22]. However, substituent size alone does not determine the extent of local crowding; the relative orientations of neighboring groups also matter. Intramolecular hydrogen bonding can favor particular substituent orientations and limit the conformational adjustments available to relieve crowding. An attractive interaction at one site may therefore promote unfavorable contacts elsewhere within the molecule.

Here, we examine this possibility in 1,3,5‐tris(methylamino)‐2,4,6‐trinitrobenzene (3) and four related compounds (Figure 1B). In compound **3**, intramolecular N─H···O hydrogen bonds may constrain the orientations of the methylamino groups, bringing their methyl groups into unfavorable proximity to neighboring nitro groups. Benzene ring puckering may provide a means of accommodating this local crowding. By comparing compounds that differ in hydrogen‐bond donor availability and substituent size, we investigate the relationship between hydrogen bonding, substituent orientation, and ring conformation.

## 2 Results and Discussion

The syntheses of the puckered benzene derivative and its comparative analogues are outlined in Figure 2. Starting from phloroglucinol, nitration in a KNO_3_/sulfuric acid system yielded 1,3,5‐trinitrobenzene‐2,4,6‐triol (**1**). Subsequent alkylation with trimethyl orthoformate produced 1,3,5‐trimethoxy‐2,4,6‐trinitrobenzene (**4**). Amination of compound **4** with aqueous ammonia and methylamine afforded TATB (**2**) and the central molecule of this study, 1,3,5‐tris(methylamino)‐2,4,6‐trinitrobenzene (**3**), respectively. Finally, nitration of **3** using a TFA/HNO_3_ system successfully yielded the fully nitrated analogue, 1,3,5‐trinitro‐2,4,6‐tris(methylnitramino)benzene (**5**). The NMR spectra of compounds 1, 3, 4, and 5 and the IR spectra of compounds 1–5 are provided in Figures S1–S9, respectively.

**FIGURE 2:**
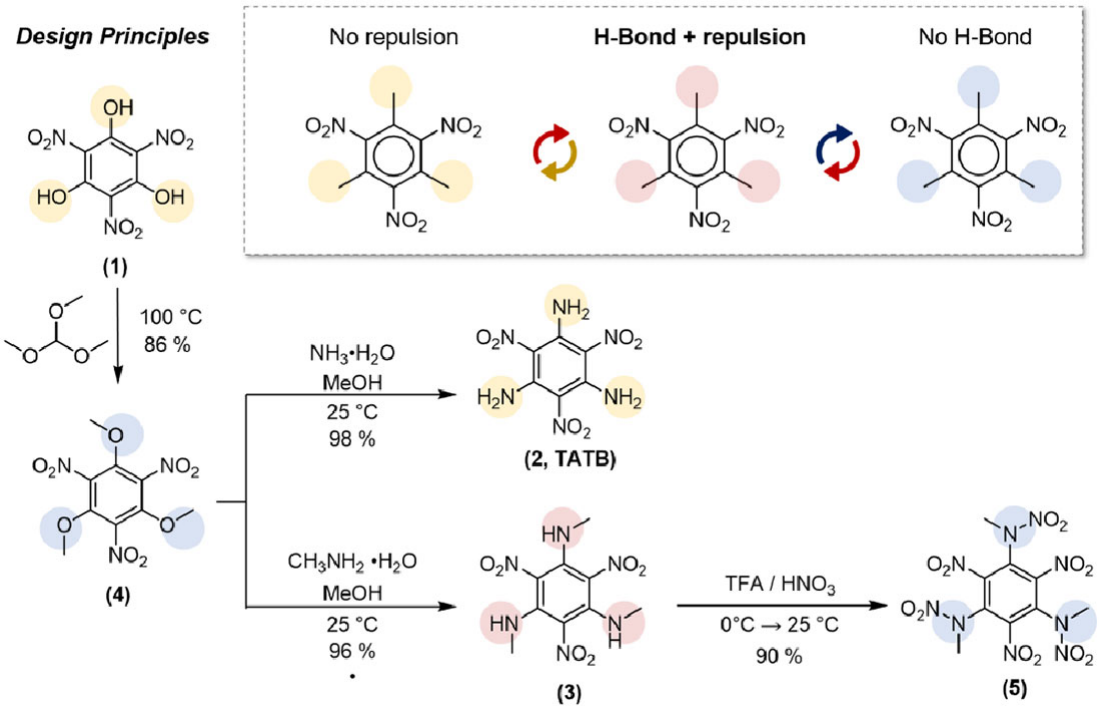
Synthetic routes and design principles of central compound 3 and its structural analogues.

Compounds **1**–**5** provide a structurally related series for examining the proposed role of hydrogen bonding in local steric crowding. In compound **3**, the methylamino groups contain both an N─H hydrogen‐bond donor and a methyl group, while adjacent nitro groups provide hydrogen‐bond acceptors. Intramolecular N–H···O interactions may therefore influence the orientations of the methylamino groups and their contacts with neighboring nitro groups [23].

Compounds **1** and **2** lack the methyl groups present in **3** but retain O–H or N–H donors capable of forming intramolecular hydrogen bonds with adjacent nitro groups. Compounds **4** and **5** retain methyl groups but lack these O–H and N–H donors. Compound **5** additionally contains larger methylnitramino substituents. These comparisons allow us to examine how changes in hydrogen‐bond donor availability and substituent structure are associated with benzene ring puckering.

To examine the ring conformations of compounds **1**–**5** and their relationship to intramolecular interactions, single crystals were grown via slow solvent evaporation and analyzed by X‐ray diffraction. As shown in Figure 3A,B, the benzene skeleton of compound **3** adopts a distinctly puckered, boat‐shaped conformation, with twist angles of 29.6° on either side. By comparison, the benzene rings of compounds **1**, **2**, **4**, and **5** exhibit no appreciable ring puckering, maintaining nearly planar conformations (with torsion angles of approximately 0°). Notably, the C─C bond lengths within the benzene rings of these five compounds are highly consistent, ranging from 1.384 to 1.447 Å, indicating no significant skeletal bond alternation. Crystallographic data, molecular structures, packing diagrams, and selected bond lengths and angles for compounds 3 and 5 are provided in Figures S10 and S11 and Tables S1–S3.

**FIGURE 3:**
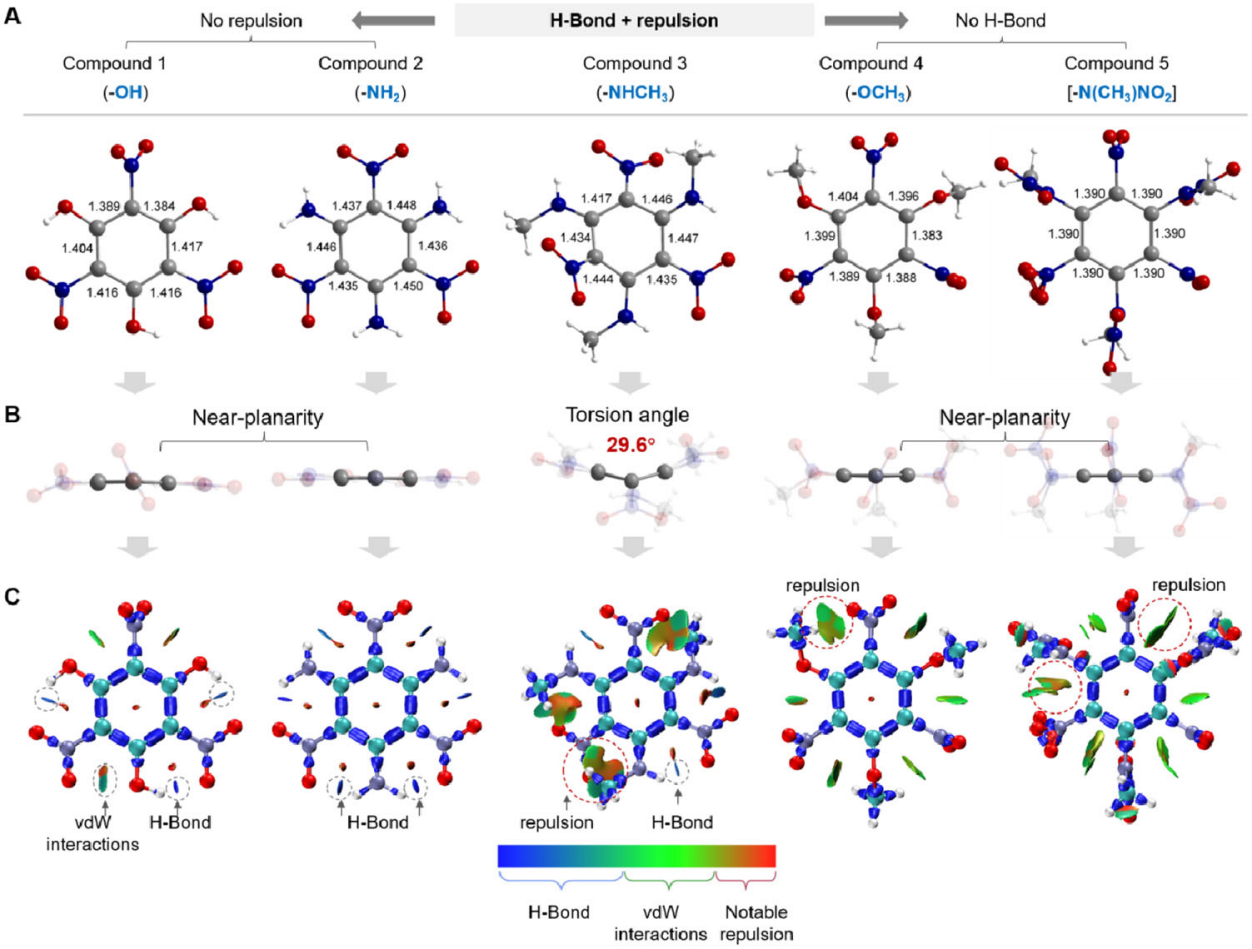
Comprehensive structural and theoretical comparison of compounds 1–5. (A) Molecular structures and schematic representation of the target interactions. (B) Single‐crystal X‐ray structures highlighting key intra‐ring C─C bond lengths (top) and out‐of‐plane torsion angles (bottom). (C) IRI analysis plots visually illustrating the intramolecular hydrogen‐bonding networks (blue/cyan isosurfaces) and localized steric repulsion regions (red/yellow isosurfaces).

Geometrical analysis of the experimental crystal structures using Mercury identified intramolecular O─H···O or N─H···O hydrogen bonds in compounds **1**–**3**. The intramolecular hydrogen‐bond arrangements are illustrated in Figures S12–S14, and the corresponding geometrical parameters are summarized in Tables S4–S6. Specifically, all three –NH groups in compound **3** participate in hydrogen bonding with lengths of 1.90, 1.95, and 1.95 Å, slightly longer than those observed in compound **1** (1.87, 1.75, and 1.86 Å) and compound **2** (1.80, 1.84, 1.95, 1.69, 1.82, and 1.77 Å). Interaction Region Indicator (IRI) analysis was performed using Multiwfn based on electron densities obtained from single‐point calculations at the B3LYP/6‐311++G(d,p) level using the experimental crystal geometries [24, 25, 26]. Further computational details are provided in the Supporting Information. Furthermore, as shown in Figure 3C, IRI analysis visually corroborates a pronounced steric repulsion between the methyl and nitro groups in compound **3**. By contrast, in the absence of hydrogen‐bonding attraction, the corresponding repulsion in compound **4** is significantly weaker. Strikingly, even in compound **5**, which possesses much bulkier substituents, the intramolecular group repulsion is markedly less intense than that in **3**. Additional localized orbital locator‐π (LOL‐π) analysis of compounds 1–5 is provided in Figure S15 and Section 6.3. Variable‐temperature FTIR and UV–vis measurements were also performed for compounds **1**–**5** to examine their spectroscopic responses to heating (Figures S18–S22).

Together, the crystallographic observations and IRI analysis support an interpretation in which intramolecular hydrogen bonding constrains substituent orientations in compound **3**, contributing to local crowding between methyl and nitro groups. The boat‐shaped ring conformation may help accommodate these contacts. The nearly planar rings of the related compounds further suggest that the combination of hydrogen‐bonding interactions and substituent arrangement, rather than substituent size alone, is relevant to the observed puckering.

In addition to their structural features, polynitroarenes represent a critical class of energetic materials. For example, the quintessential explosive TNT is formulated as 2,4,6‐trinitrotoluene (Figure 4A). In fact, compound **2** is the well‐known insensitive high explosive TATB, and the energetic properties of compound 1 have also been extensively investigated. However, as shown in Figure 4C, the energetic performance of compound **5**, a highly nitrated derivative, remains unexplored. Therefore, we evaluated the energetic and physicochemical properties of compound **5** and systematically compared them with those of TNT and our previously reported 1, 3, 5‐trinitro‐2, 4, 6‐trinitroaminobenzene (TNTNB) (since **5** can be easily synthesized via the methylation of TNTNB, Figure 4B) [5, 27]. The DSC curves of compounds 3 and 5 are shown in Figures S16 and S17. The isodesmic reactions and thermochemical data used to calculate their heats of formation are provided in Schemes S1 and S2 and Table S7, respectively (Supporting Information). Compound **5** exhibits a crystal density of 1.692 g/cm^3^ and a calculated detonation velocity of 7838 m/s. Its decomposition temperature, impact sensitivity, and friction sensitivity are 202°C, 9 J, and 96 N, respectively (Figure 4 and Table S8). Compared with TNT, compound **5** has a higher calculated detonation velocity but a lower decomposition temperature and lower impact and friction thresholds. Relative to TNTNB, it shows a lower calculated detonation velocity together with a higher decomposition temperature and higher mechanical sensitivity thresholds.

**FIGURE 4:**
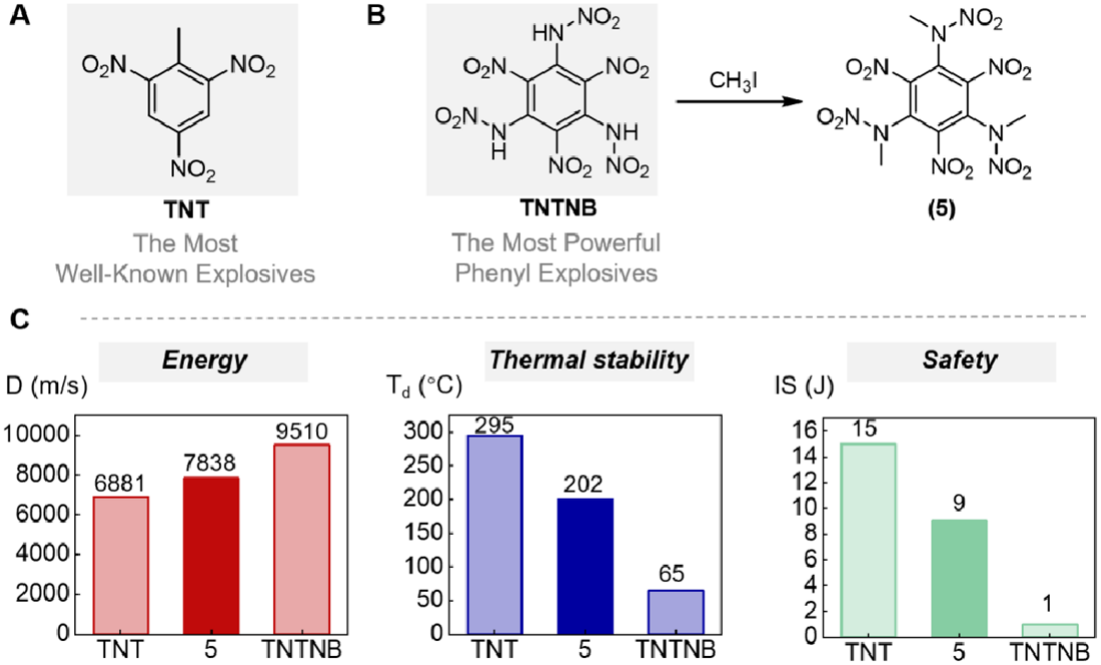
Evaluation of the physicochemical and energetic properties of compound 5 in comparison with TNT and TNTNB. (A) Chemical structure of TNT as a classic benchmark. (B) Synthetic derivation of compound **5** via the methylation of TNTNB. (C) Comparative histograms highlighting the detonation velocity (D), thermal decomposition temperature (T_d_), and impact sensitivity (IS) of TNT, compound **5**, and TNTNB.

## 3 Conclusion

In summary, we have presented an intriguing phenomenon where benzene ring distortion is driven by hydrogen‐bonding‐induced small‐group repulsion. By systematically eliminating hydrogen‐bond donors and reducing steric bulk in a series of structural analogues (**1**–**5**), we elucidated the synergistic mechanism between intramolecular hydrogen bonding and localized steric repulsion. Our findings show that pronounced benzene ring puckering can occur in a system bearing relatively small substituents, highlighting the importance of their conformational arrangement. Furthermore, the fully nitrated derivative, compound **5**, was identified as a potential energetic material featuring a desirable balance between detonation performance and stability. This work not only provides fresh fundamental insights into the structural chemistry of distorted benzenes but also expands the structural diversity of polynitroarenes as promising explosive materials.

## Author Contributions

**Chaofeng Zhao**: validation, writing – original draft, methodology, conceptualization, visualization, formal analysis, data curation, project administration, investigation, supervision. **Qi Sun**: conceptualization, methodology, formal analysis, visualization, funding acquisition, writing – review and editing, data curation, investigation. **Ning Ding**: writing – original draft, writing – review and editing, visualization, formal analysis. **Xiaoting Ren**: resources, project administration. **Jinxuan He**: resources. **Shenghua Li**: writing – review and editing, funding acquisition, resources. **Siping Pang**: resources, funding acquisition.

## Funding

This work was supported by the National Natural Science Foundation of China (Grant Nos. 22575026, 22305020, 22235003, and 22375022) and the Open Research Fund Program of the National Key Laboratory of Aerospace Chemical Power (Grant No. NKLACP120251A19).

## Conflicts of Interest

The authors declare no conflicts of interest.

## Supporting information




**Supporting File**: advs78352‐sup‐0001‐SuppMat.pdf.

## Data Availability

The data that support the findings of this study are available from the corresponding author upon reasonable request.
