# Supplementary material for: H‐Bonding‐Induced Repulsion Between Small Groups Toward a Highly Puckered Benzene
Source: Adv Sci (Weinh). 2026 Oct 18:e78352. Online ahead of print. doi: 10.1002/advs.78352 (PMC13676994; doi:10.1002/advs.78352)
Supplement: Supplementary file 1 [file ADVS-9999-e78352-s001.pdf]

Supporting Information

H-bonding-induced repulsion between small groups towards a highly puckered benzene

Chaofeng Zhao,[a] Qi Sun,[a] Ning Ding,*[a] Xiaoting Ren,[b] Jinxuan He,[b] Shenghua Li,*[a] and Siping Pang[a]*

[a] School of Materials Science & Engineering, Beijing Institute of Technology, Beijing 100081, China

[b] Hubei Institute of Aerospace chemical Technology, Xiangyang 12 441002

E-mail: sunqi@bit.edu.cn; dingning@bit.edu.cn; lishenghua@bit.edu.cn

Table of Contents

1. General methods
2. Safety precautions
3. Experimental procedures
4. Spectrum analysis
5. Crystal structures
6. Computational details
7. Thermal stability
8. Energetic properties
9. variable-temperature FTIR and UV-vis measurements

1. General Methods

All reagents were purchased from Energy Chemical of analytical grade and were used as supplied, if not stated otherwise. The melting points and decomposition temperatures were obtained on a differential scanning calorimeter (Mettler Toledo DSC823e) at a scan rate of 10 °C/min in closed Al containers with a nitrogen flow of 50 mL/min.

2. Safety Precautions

All the new compounds in this manuscript are powerful explosives, and should be handled with extreme care using the best safety practices.

3. Experimental procedures

1,3,5-trinitrobenzene-2,4,6-triol (**1**): At 0 °C, KNO_3 (13 g) was added in batches to 112 mL of 98% sulfuric acid, followed by stirring for 30 minutes. Then, phloroglucinol (5 g) was added in batches to the reaction system and stirred at 0 °C for 2 hours. The reaction system was poured into 300 g of crushed ice, and the solid was filtered. The filter cake was rinsed three times with ice water and air-dried to obtain 1,3,5-trinitrobenzene-2,4,6-triol (**1**) (8.6 g, 83%). ^{1}H NMR (DMSO-d_6): 7.57 ppm. IR (KBr): =(detect) 3645, 3576, 3133, 2885, 2605, 1633, 1586, 1533, 1467, 1420, 1363, 1319, 1212, 1178, 914, 810, 794, 763, 722, 697, 637 cm^{-1}.

1,3,5-trimethoxy-2,4,6-trinitrobenzene (**4**): A solution of **1** (5 g) in 30 mL trimethy orthoformate was heated to 100 °C and refluxed for 5 hours. Distillation under reduced pressure and purification via recrystallization, 1,3,5-trimethoxy-2,4,6-trinitrobenzene (**4**) (4.9 g, 86%) was obtained. ^{1}H NMR (DMSO-d_6): 3.98 ppm. IR (KBr): =(detect) 3415, 2957, 2878, 1595, 1539, 1457, 1401, 1353, 1259, 1196, 1128, 1106, 938, 883, 841, 737, 718, 658, 633, 573 cm^{-1}.

TATB (**2**): A solution of **4** (3 g) in methanol (100 mL), add 15 mL of ammonia water at 25 °C and stir for 2 hours. Filter the mixture, rinse the filter cake with methanol three times, and dry to obtain TATB (**2**) (2.5 g, 98%). IR (KBr): =(detect) 3321, 3226, 1604, 1566, 1447, 1222, 1173, 1029, 784, 725, 694 cm^{-1}.

1,3,5-tris(methylamino)-2,4,6-trinitrobenzene (**3**): A solution of **4** (3 g) in methanol (100 mL), add 15 mL of aqueous methylamine at 25 °C and stir for 2 hours. Filter the mixture, rinse the filter cake with methanol three times, and dry to obtain 1,3,5-tris(methylamino)-2,4,6-trinitrobenzene (**3**) (2.5 g, 98%).^{1}H NMR (DMSO-d_6): 10.27, 3.08 ppm. IR (KBr): =(detect) 3448, 3079, 2810, 2462, 2126, 1612, 1482, 1406, 1334, 1236, 1195, 1144, 1083, 1033, 980, 922, 902, 777, 758, 710, 648, 602, 538 cm^{-1}.

1,3,5-trinitro-2,4,6-tris(methylnitramino)benzene (**5**): A solution of **3** (300 mg) in trifluoroacetic acid (4 mL) was stirred at 0 °C and treated by dropwise addition of Fuming nitric acid (1 mL) and the mixture was stirred for 3 hours at room temperature. Pour the reaction solution into ice, and a solid precipitates. Filter to obtain 1,3,5-trinitro-2,4,6-tris(methylnitramino)benzene (**5**) (391 mg, 90%). ^{1}H NMR (CD_3CN): 2.41 ppm. IR (KBr): =(detect) 3413, 2889, 1554, 1420, 1353, 1282, 1147, 1117, 1013, 927, 849, 800, 752, 711, 614, 562 cm^{-1}.

4. Spectrum Analysis

4.1 NMR spectra.

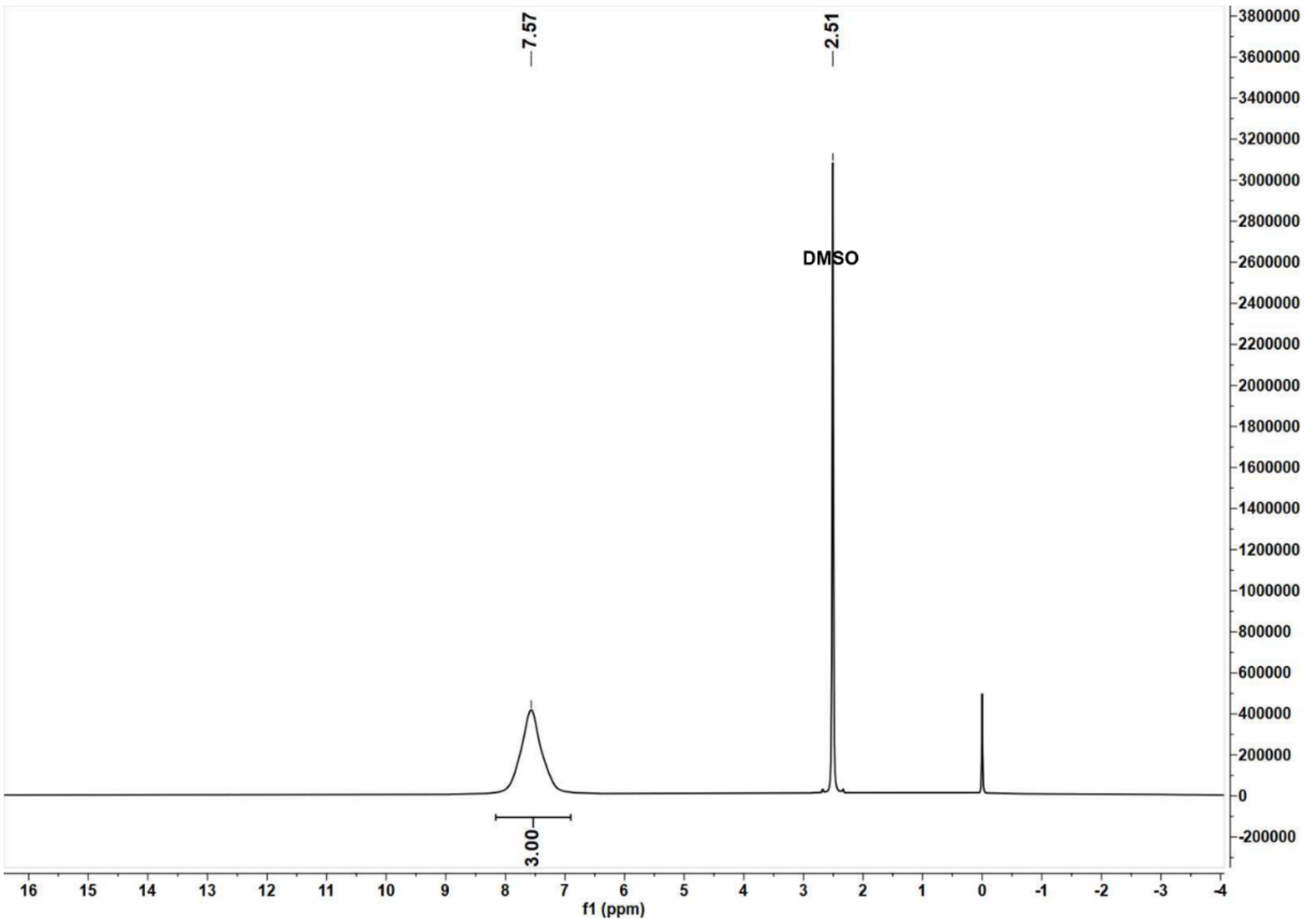

Figure S1. ^{1}H NMR of **1** in DMSO-d$_6$.

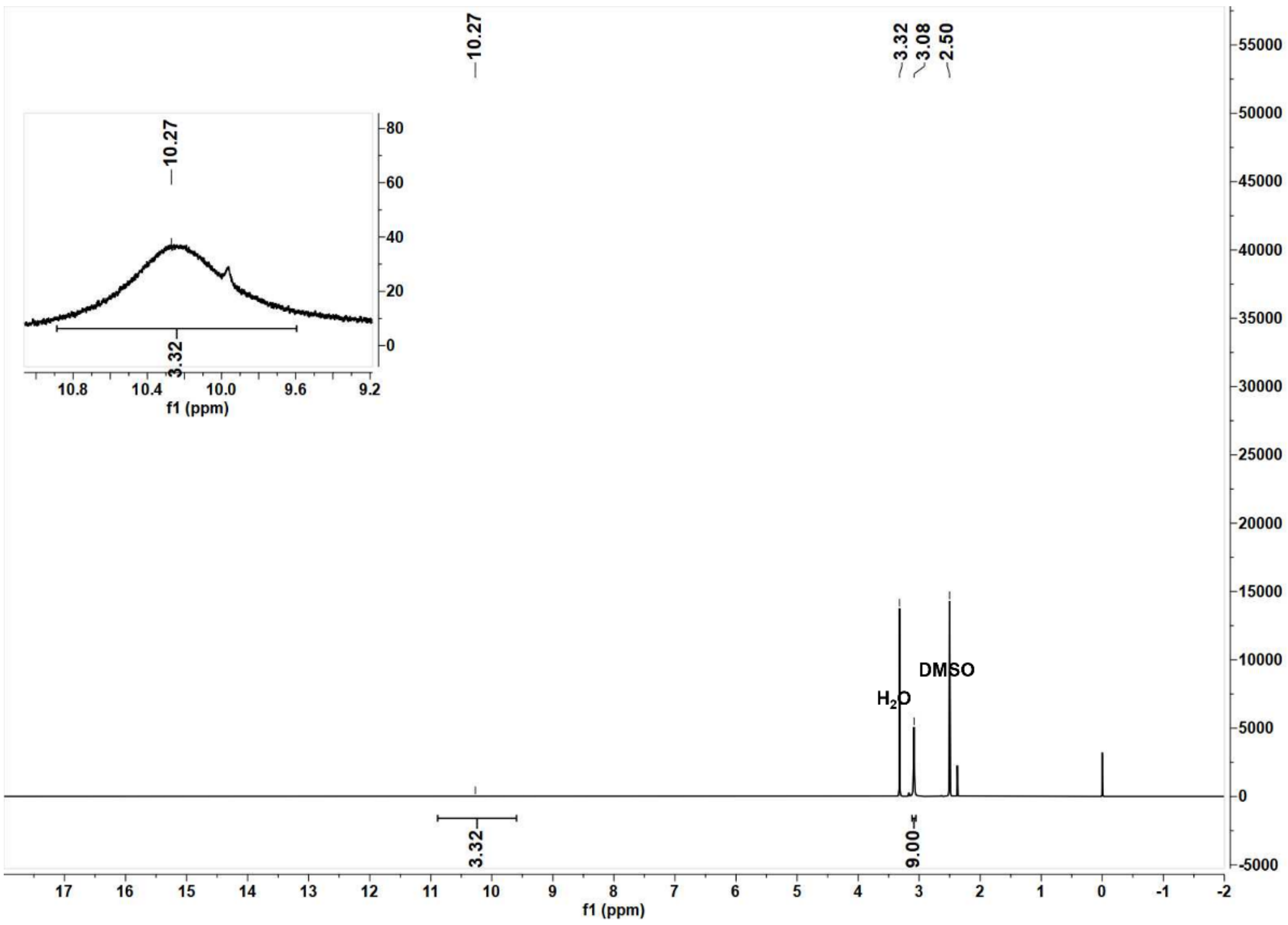

Figure S2. ^{1}H NMR of **3** in DMSO-d$_6$.

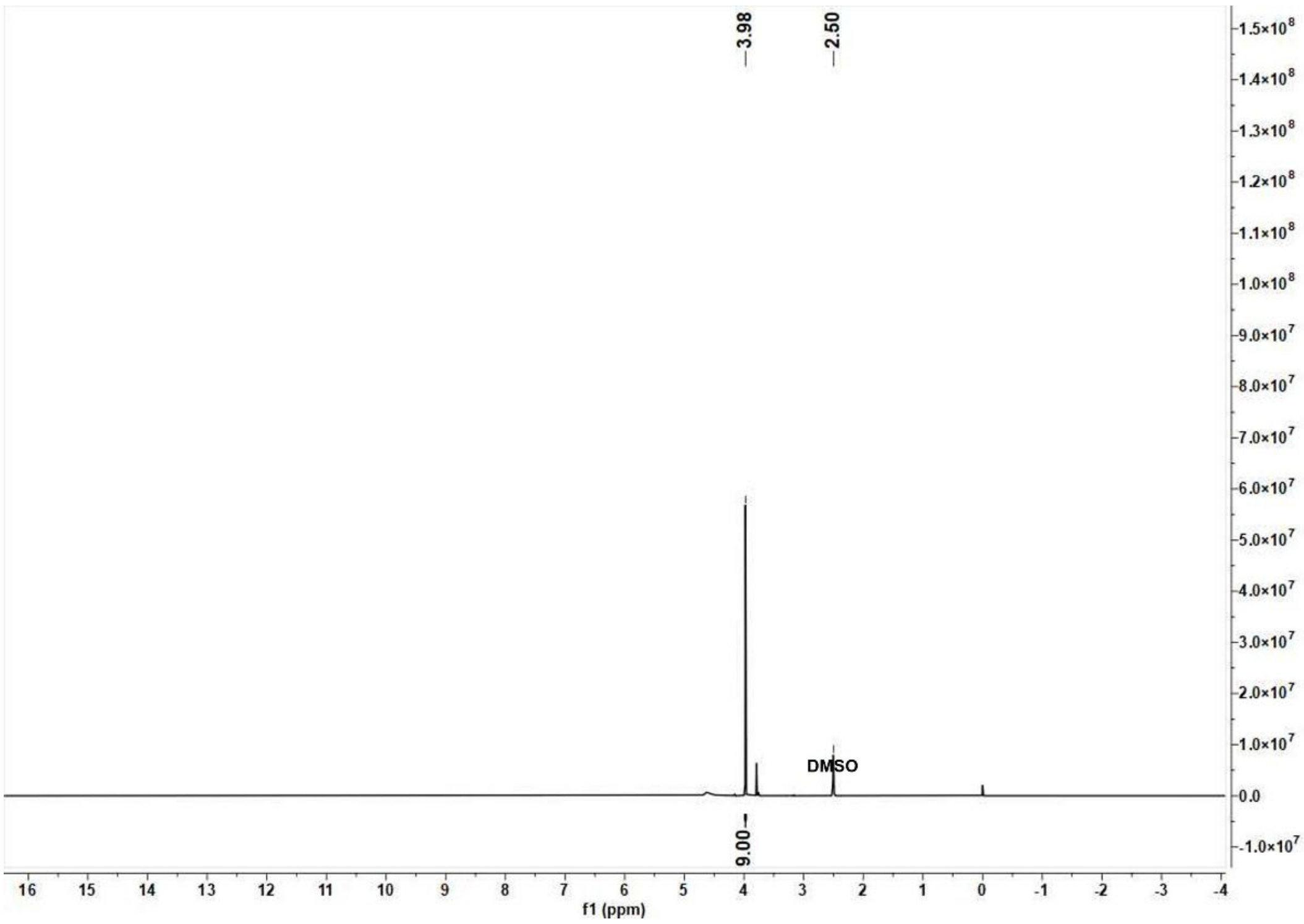

Figure S3. ^{1}H NMR of **4** in DMSO-d$_6$.

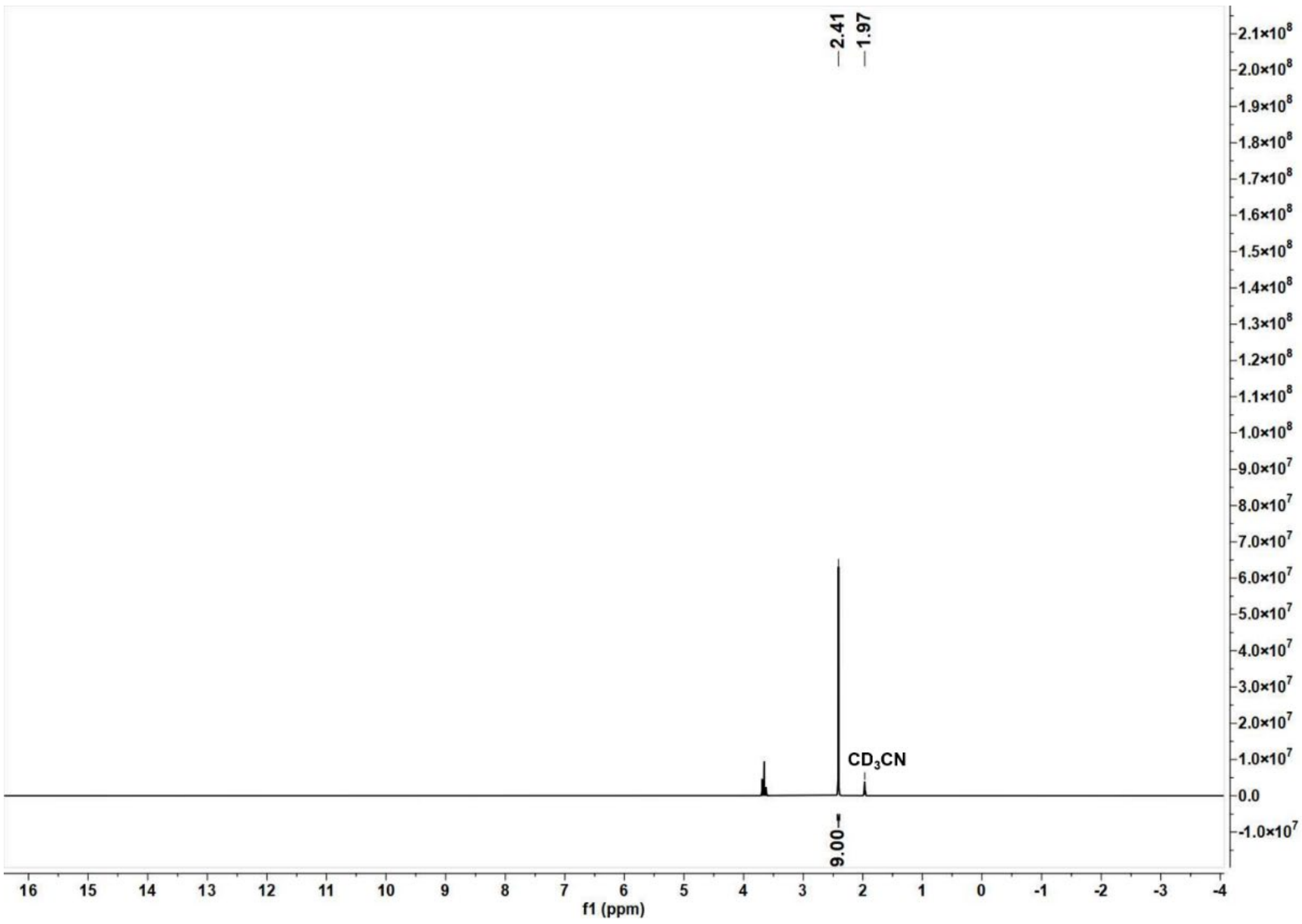

Figure S4. ^{1}H NMR of **5** in CD_3CN.

4.2 IR Spectrum.

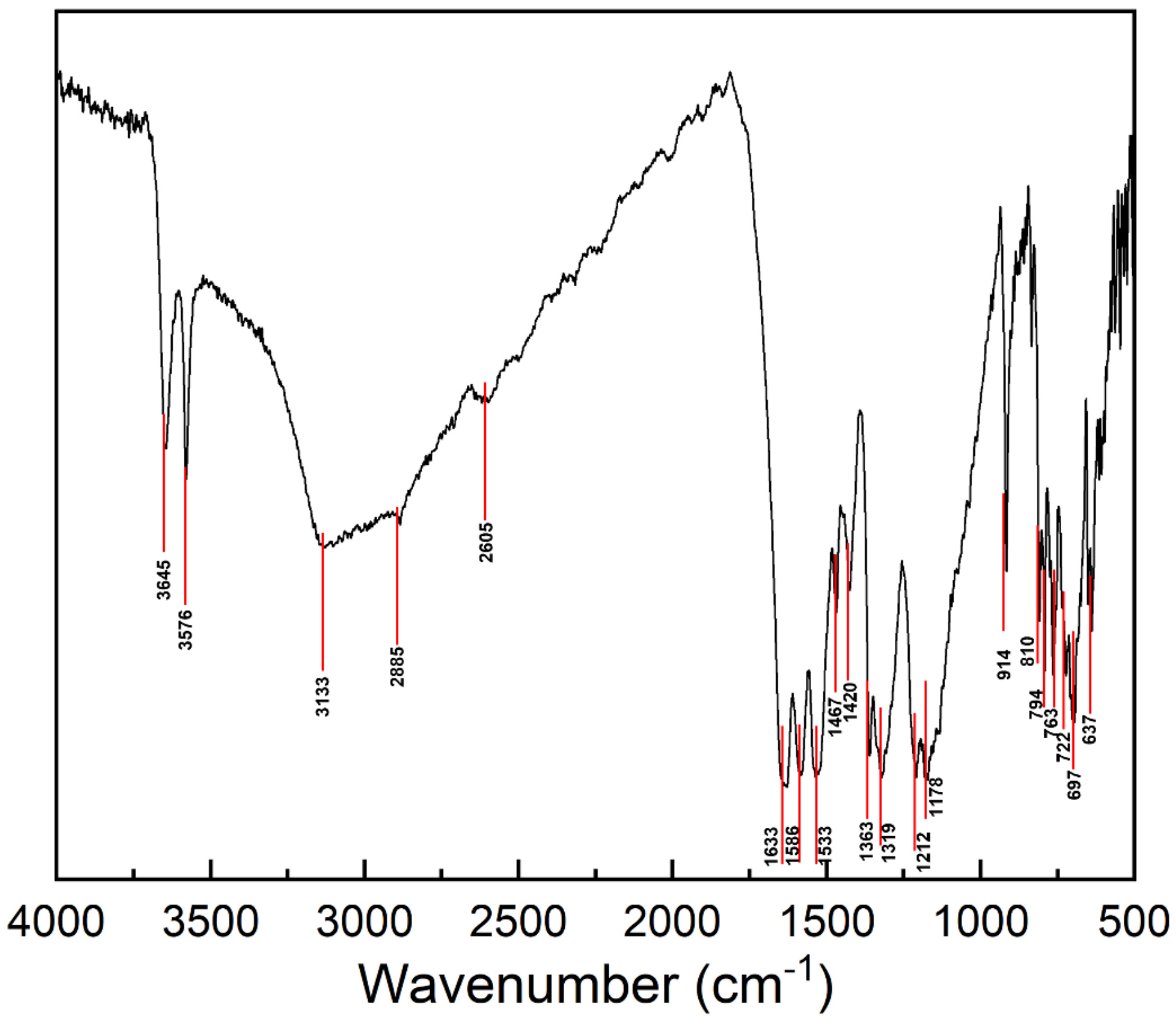

Figure S5. IR spectrum of **1**.

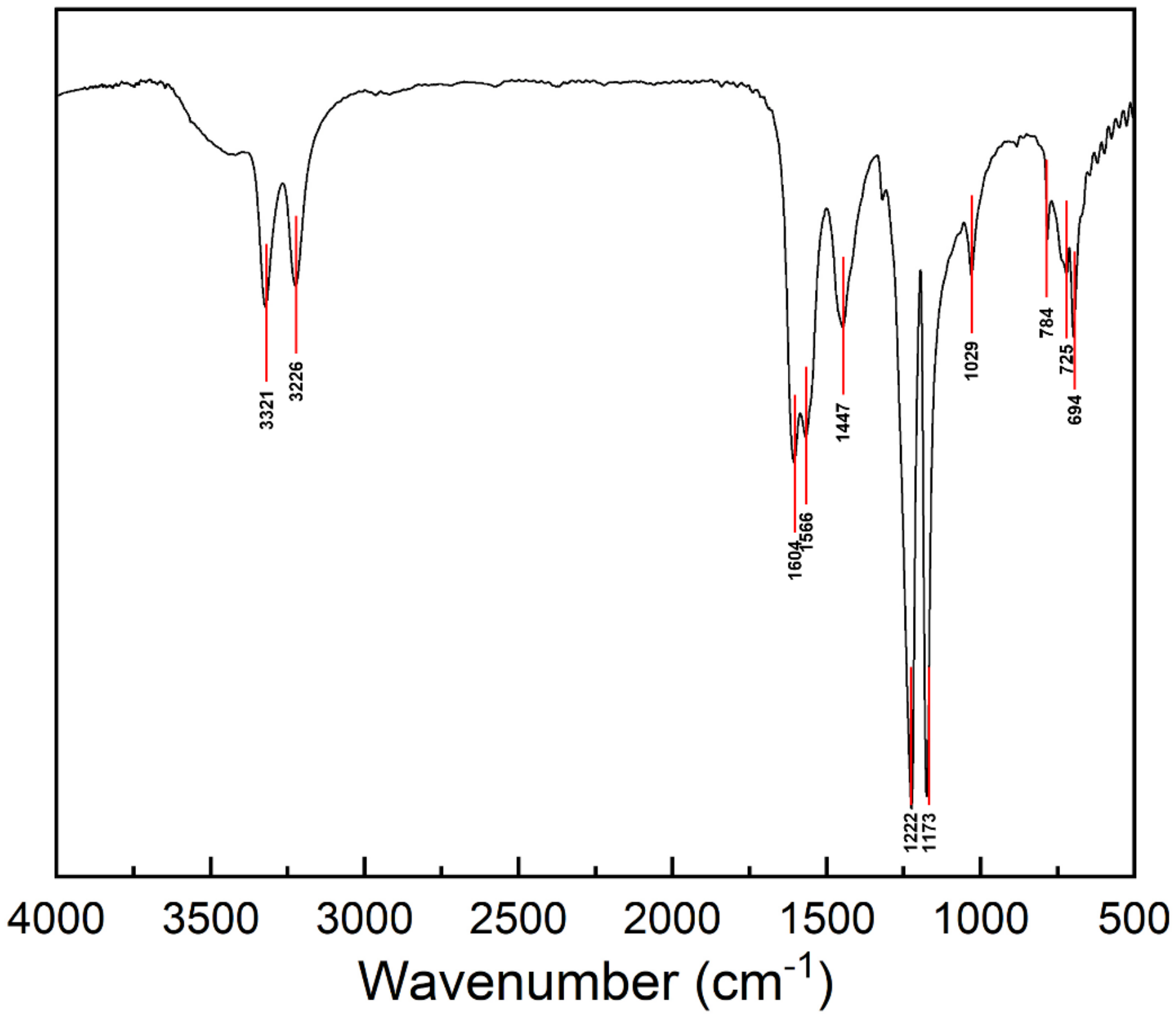

Figure S6. IR spectrum of **2**.

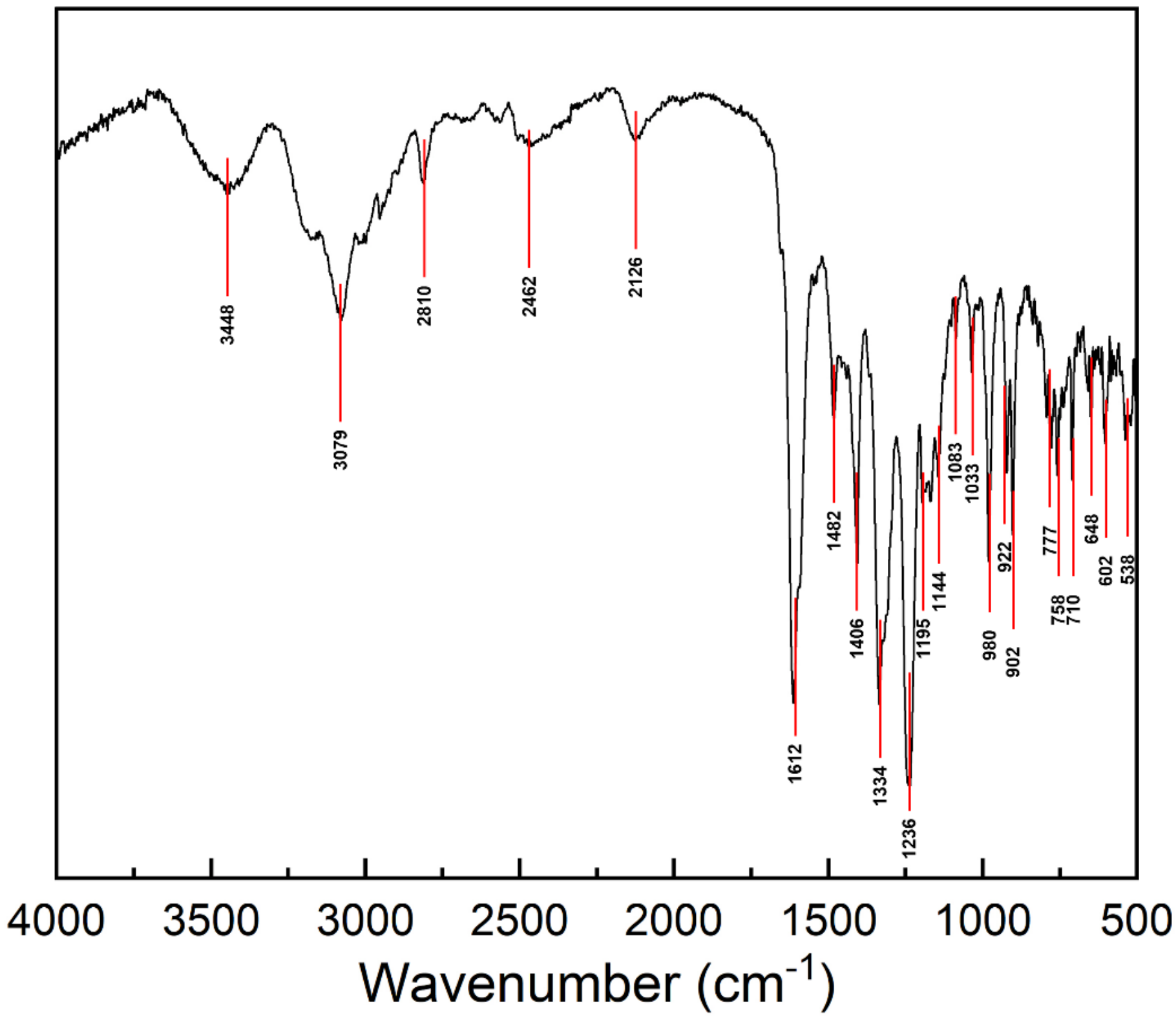

Figure S7. IR spectrum of **3**.

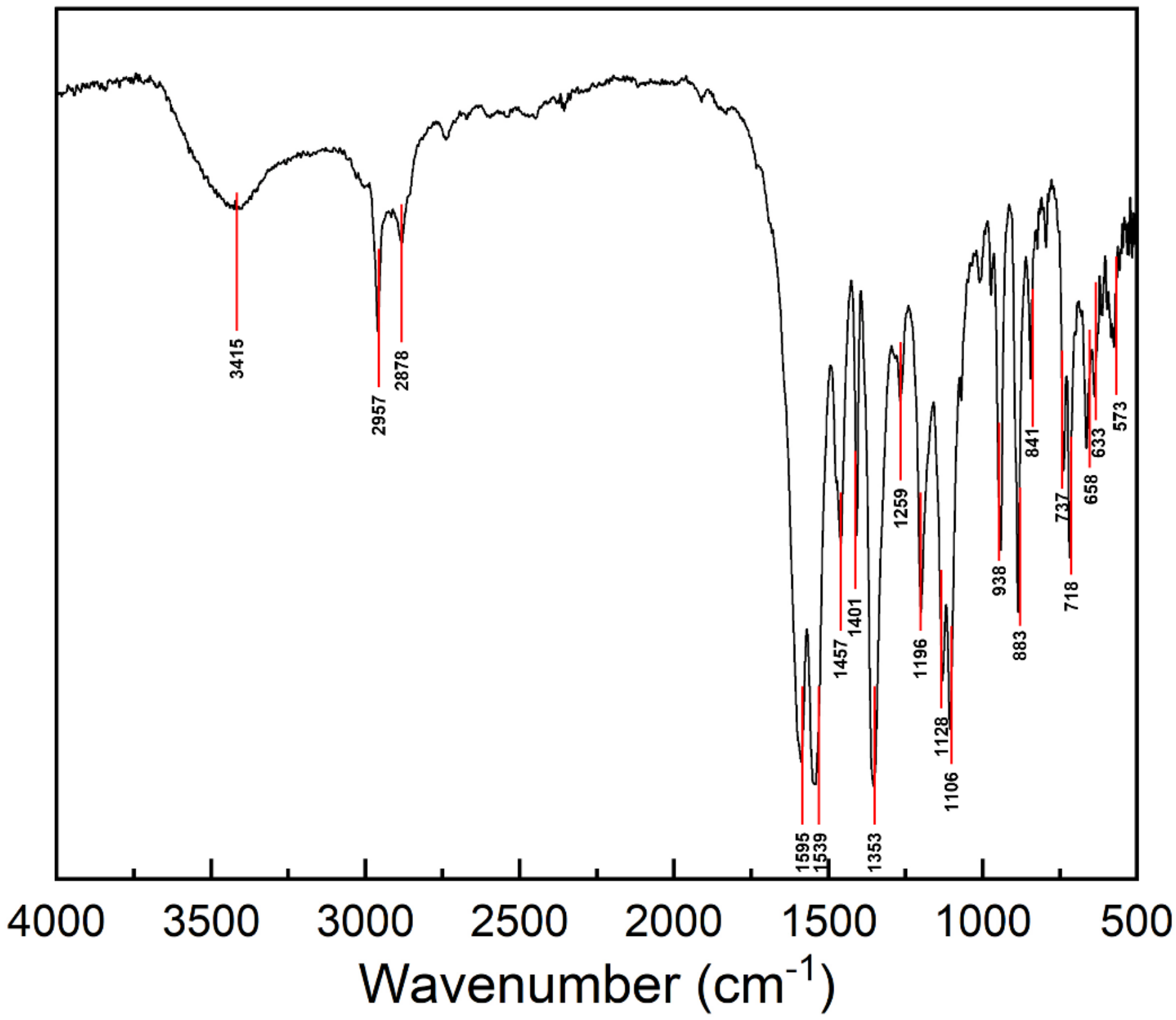

Figure S8. IR spectrum of **4**.

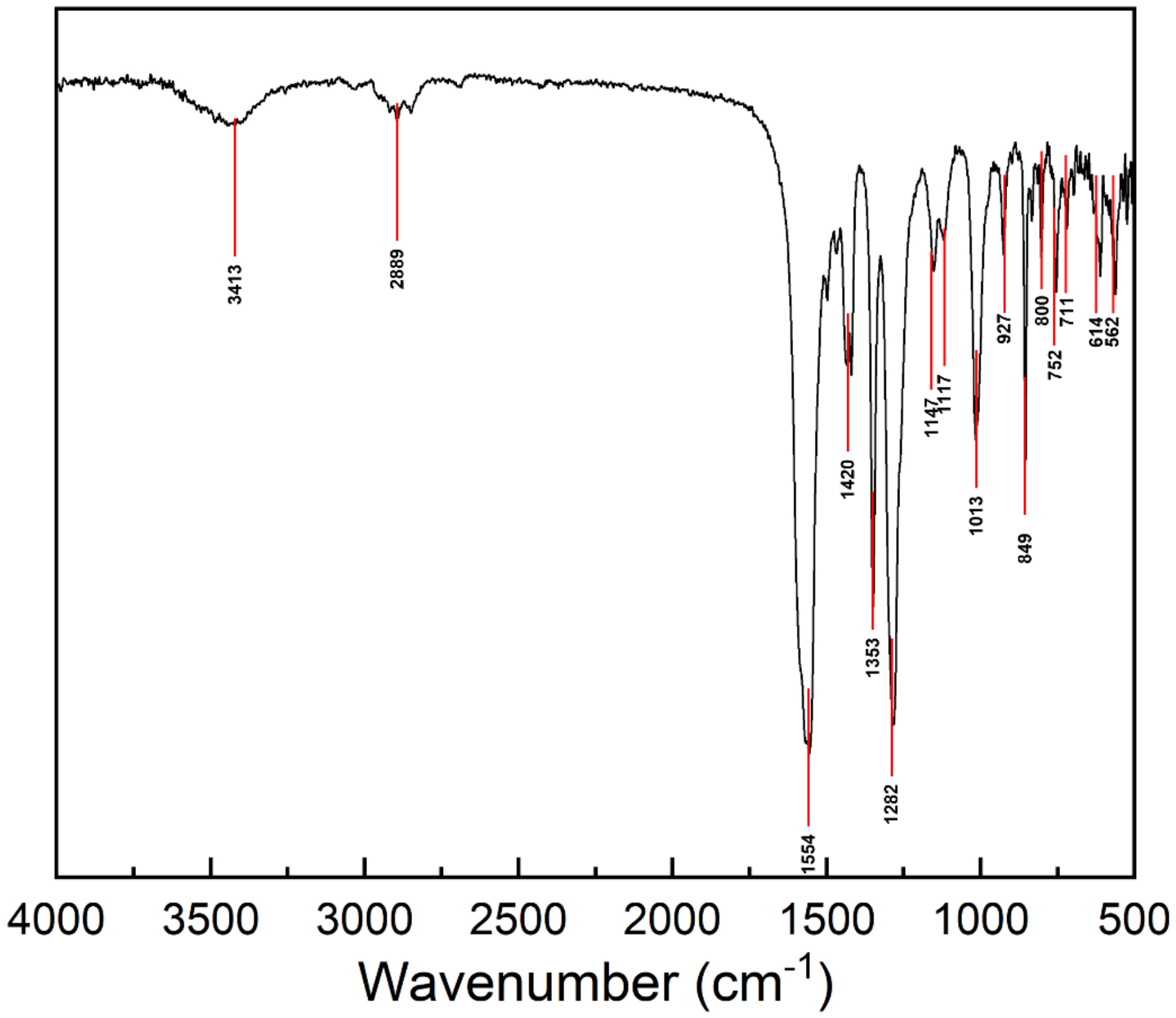

Figure S9. IR spectrum of **5**.

5. Crystal Structures

5.1 X-ray crystallography details

A yellow block crystal (**3**) of dimensions 0.20×0.20×0.20 mm^3 was mounted on an Enraf-Nonius CAD4 four-circle diffractometer using graphite-monochromated Mo Ka radiation (λ = 0.71073 Å) at 296 K. Corrections for Lorentz and polarization effects and for absorption (ψ scan) were applied. The structure was solved by direct methods using SHELXL-2014/7 and refined by full-matrix least-squares calculation on F2 with SHELXL-2014/7. All non-hydrogen atoms were refined anisotropically. All hydrogen atoms were placed in calculated positions and were assigned fixed isotropic thermal parameters at 1.2 times the equivalent isotropic U of the atoms to which they were attached and allowed to ride on their respective parent atoms. The contributions of these hydrogen atoms were included in the structure-factor calculations.

A colorless block crystal (**5**) of dimensions 0.20×0.20×0.20 mm^3 was mounted on an Enraf-Nonius CAD4 four-circle diffractometer using graphite-monochromated Cu Ka radiation (λ = 1.54184 Å) at 293 K. Corrections for Lorentz and polarization effects and for absorption (ψ scan) were applied. The structure was solved by direct methods using Olex2 1.5 and refined by full-matrix least-squares calculation on F2 with Olex2 1.5. All non-hydrogen atoms were refined anisotropically. All hydrogen atoms were placed in calculated positions and were assigned fixed isotropic thermal parameters at 1.2 times the equivalent isotropic U of the atoms to which they were attached and allowed to ride on their respective parent atoms. The contributions of these hydrogen atoms were included in the structure-factor calculations.

Table S1. Crystallographic data for **3** and **5**.

Compound	**3**	**5**
Formula	$C_9H_{12}N_6O_6$·ACN	$C_9H_9N_9O_{12}$
M_w	341.3	435.25
Crystal system	triclinic	triclinic
Space group	P -1	P 1
a [Å]	8.604 (7)	9.6546(8)
b [Å]	10.113 (9)	9.9543(8)
c [Å]	10.169 (8)	11.0717(6)
α[°]	99.401 (19)	88.571(6)
β[°]	112.862 (18)	67.059(7)
γ[°]	105.891 (18)	62.584(8)
V [$Å^3$]	747.0 (11)	854.15(13)
Z	2	2
T [K]	296	293
λ [Å]	0.71073	1.54184
P_{calcd}[g cm^{-3}]	1.517	1.692
μ [mm^{-1}]	0.125	1.410
F(000)	356.0	444
θ range[°]	2.198-25.229	4.416-76.085
Index ranges	$-10 \leq h \leq 10$<br>$-12 \leq k \leq 11$<br>$-12 \leq l \leq 12$	$-11 \leq h \leq 12$<br>$-12 \leq k \leq 12$<br>$-9 \leq l \leq 13$
Data/restraints/parameters	2691/0/221	4964/3/547
GOF on F2	1.009	1.033
R[F^2> 2σ(F^2)]	0.0638 (1410)	0.0542 (4747)
wR(F^2)	0.2039 (2691)	0.1658 (4964)

5.2 Bond lengths and angles

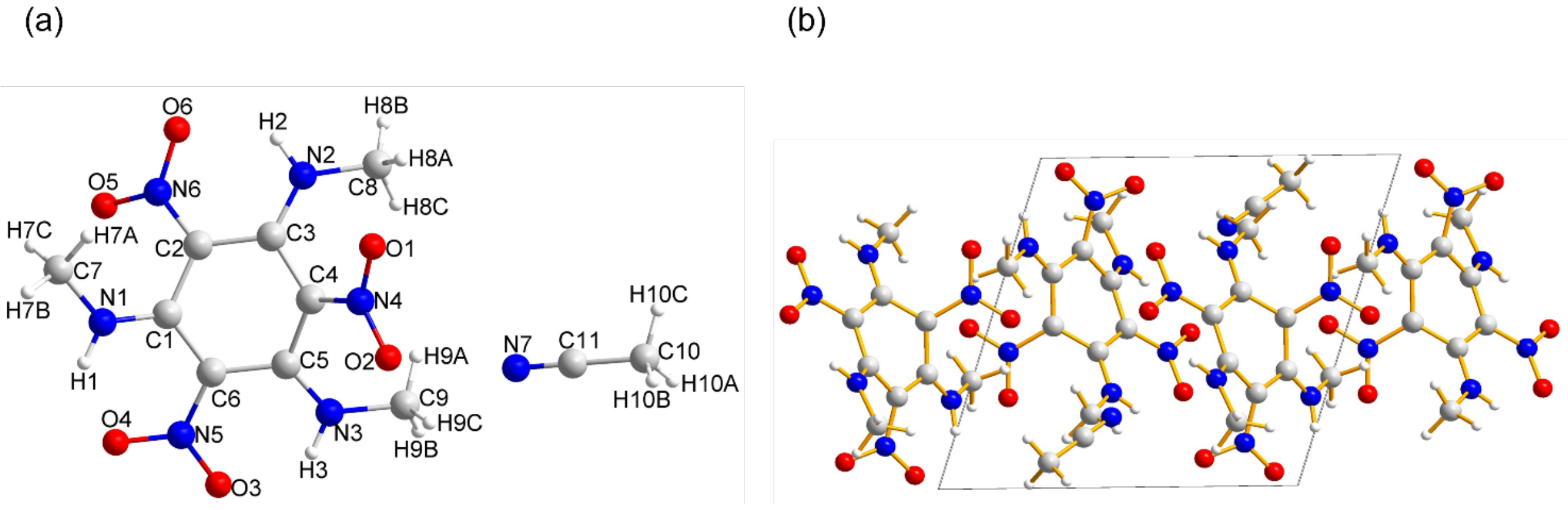

Figure S10. (a) Crystal structure of **3**; (b) Packing diagram of **3**.

Table S2. Bond lengths (Å) and bond angles (°) for **3**.

Bond lengths		
C1 N1 1.306(5)	C7 H7A 0.9600	N3 H3 0.8600
C1 C2 1.446(5)	C7 H7B 0.9600	N4 O2 1.243(4)
C1 C6 1.447(5)	C7 H7C 0.9600	N4 O1 1.247(4)
C2 N6 1.388(5)	C8 N2 1.454(5)	N5 O4 1.255(4)
C2 C3 1.418(5)	C8 H8A 0.9600	N5 O3 1.257(4)
C3 N2 1.318(4)	C8 H8B 0.9600	N6 O5 1.240(4)
C3 C4 1.433(5)	C8 H8C 0.9600	N6 O6 1.254(4)
C4 N4 1.404(5)	C9 N3 1.454(5)	C10 C11 1.419(9)
C4 C5 1.443(5)	C9 H9A 0.9600	C10 H10A 0.9600
C5 N3 1.309(5)	C9 H9B 0.9600	C10 H10B 0.9600
C5 C6 1.435(5)	C9 H9C 0.9600	C10 H10C 0.9600
C6 N5 1.388(5)	N1 H1 0.8600	C11 N7 1.124(8)
C7 N1 1.448(5)	N2 H2 0.8600	
**Bond angles**		
N1 C1 C2 120.6(4)	N3 C5 C4 121.6(3)	H8A C8 H8B 109.5
N1 C1 C2 120.6(4)	C6 C5 C4 115.9(3)	N2 C8 H8C 109.5
C2 C1 C6 116.3(3)	H7B C7 H7C 109.5	C9 N3 H3 116.4
N6 C2 C3 120.0(3)	N2 C8 H8A 109.5	O2 N4 O1 121.1(3)
N6 C2 C1 117.9(3)	N2 C8 H8A 109.5	O2 N4 C4 120.0(3)
C3 C2 C1 120.1(3)	H8A C8 H8C 109.5	O1 N4 C4 118.8(3)
N2 C3 C2 122.6(3)	H8B C8 H8C 109.5	O4 N5 O3 119.0(3)

N2 C3 C4 121.4(3)	N3 C9 H9A 109.5	O4 N5 C6 120.4(3)
C2 C3 C4 115.7(3)	N3 C9 H9B 109.5	O3 N5 C6 120.6(3)
N4 C4 C3 119.2(3)	H9A C9 H9B 109.5	O5 N6 O6 119.9(3)
N4 C4 C5 119.4(3)	N3 C9 H9C 109.5	O5 N6 C2 120.9(3)
C3 C4 C5 119.2(3)	H9A C9 H9C 109.5	O6 N6 C2 119.2(3)
N3 C5 C6 122.5(3)	H9B C9 H9C 109.5	C11 C10 H10A 109.5
N5 C6 C5 121.4(3)	C1 N1 C7 127.1(3)	C11 C10 H10B 109.5
N5 C6 C1 119.7(3)	C1 N1 H1 116.4	H10A C10 H10B 109.5
C5 C6 C1 118.9(3)	C7 N1 H1 116.4	C11 C10 H10C 109.5
N1 C7 H7A 109.5	C3 N2 C8 126.2(3)	H10A C10 H10C 109.5
N1 C7 H7B 109.5	C3 N2 H2 116.9	H10B C10 H10C 109.5
H7A C7 H7B 109.5	C8 N2 H2 116.9	N7 C11 C10 178.3(7)
N1 C7 H7C 109.5	C5 N3 C9 127.1(3)	
H7A C7 H7C 109.5	C5 N3 H3 116.4	

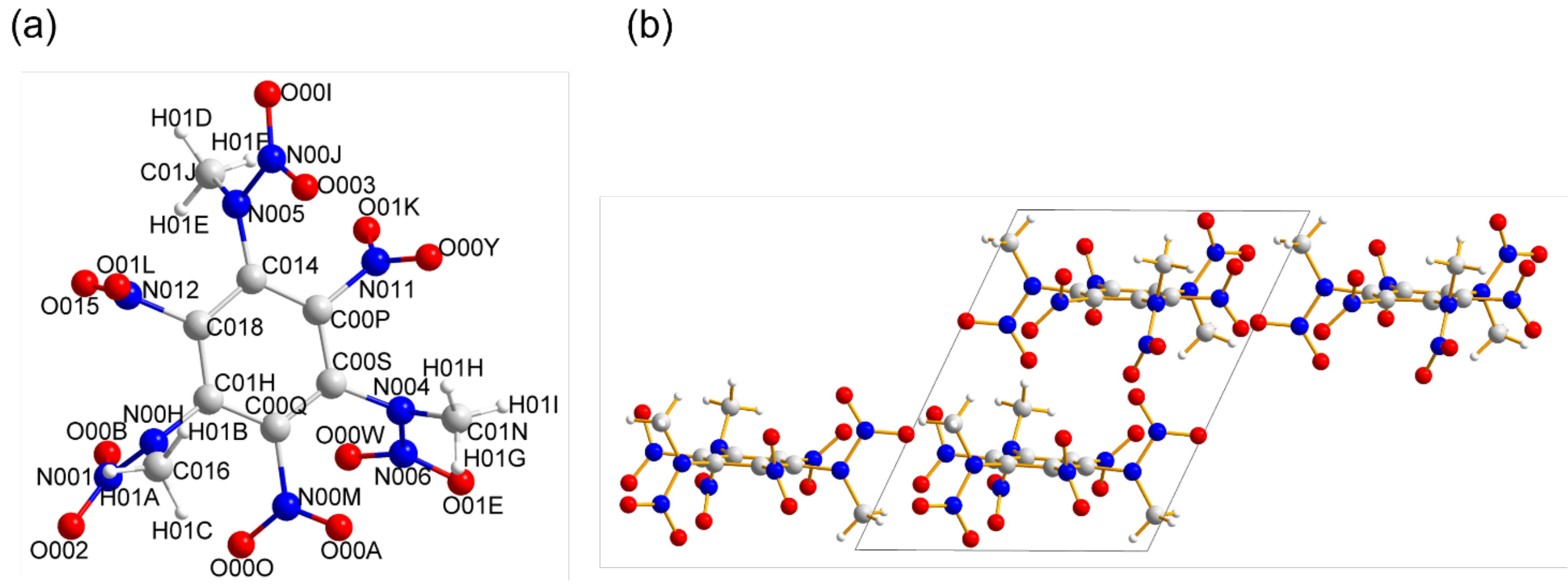

Figure S11. (a) Crystal structure of **5**; (b) Packing diagram of **5**.

Table S3. Bond lengths (Å) and bond angles (°) for **5**.

Bond lengths		
N001 O002 1.217(5)	C00F C00T 1.396(5)	C010 C01G 1.395(5)
N001 O00B 1.231(5)	C00F C01C 1.392(5)	N011 O01K 1.161(6)
N001 N00H 1.366(5)	N00G N00R 1.363(5)	N012 O015 1.191(7)
O003 N00J 1.221(5)	N00G O017 1.222(5)	N012 C018 1.471(5)
N004 N006 1.389(6)	N00H C016 1.458(5)	N012 O01L 1.195(7)
N004 C00S 1.419(5)	N00H C01H 1.413(4)	C014 C018 1.388(5)

N004 C01N 1.435(6)	O00I N00J 1.212(5)	C016 H01A 0.9600
N005 N00J 1.376(5)	N00K O00V 1.207(5)	C016 H01B 0.9600
N005 C014 1.418(4)	N00K C01C 1.474(4)	C016 H01C 0.9600
N005 C01J 1.452(6)	N00L N01D 1.381(5)	C018 C01H 1.383(5)
N006 O00W 1.206(7)	N00L C01M 1.451(6)	N019 C01O 1.445(7)
N006 O01E 1.207(6)	N00M O00O 1.226(6)	C01F H01J 0.9600
N007 C00T 1.464(5)	N00M C00Q 1.471(4)	C01F H01K 0.9600
N007 O00X 1.181(7)	C00P C00S 1.385(5)	C01F H01L 0.9600
N007 O01A 1.213(6)	C00P N011 1.467(5)	C01J H01D 0.9600
O008 N01D 1.201(5)	C00P C014 1.394(5)	C01J H01E 0.9600
N009 O00N 1.222(6)	C00Q C00S 1.387(5)	C01J H01F 0.9600
N009 O013 1.197(7)	C00Q C01H 1.395(5)	C01M H01M 0.9600
N009 N019 1.381(6)	N00R C00U 1.423(4)	C01M H01N 0.9600
O00A N00M 1.207(6)	N00R C01F 1.449(5)	C01M H01O 0.9600
O00C N00K 1.212(5)	C00T C00U 1.379(5)	C01N H01G 0.9600
N00D O01B 1.253(7)	C00U C01G 1.387(5)	C01N H01H 0.9600
N00D C01G 1.458(5)	O00Y N011 1.223(6)	C01N H01I 0.9600
N00D O01I 1.170(7)	O00Z N01D 1.210(5)	C01O H01P 0.9600
O00E N00G 1.212(5)	C010 N019 1.421(5)	C01O H01Q 0.9600
O00E N00G 1.212(5)	C010 C01C 1.383(5)	C01O H01R 0.9600
Bond angles		
O002 N001 O00B 125.5(4)	C00S C00P N011 119.3(3)	C00F C01C N00K 119.1(3)
O002 N001 N00H 117.8(4)	C00S C00P C014 122.9(3)	C010 C01C C00F 122.9(3)
O00B N001 N00H 116.7(3)	C014 C00P N011 117.8(3)	C010 C01C N00K 118.0(3)
N006 N004 C00S 115.4(4)	C00S C00Q N00M 119.7(3)	O008 N01D N00L 117.4(3)
N006 N004 C01N 118.7(4)	C00S C00Q C01H 121.7(3)	O008 N01D O00Z 126.0(4)
C00S N004 C01N 121.5(4)	C01H C00Q N00M 118.6(3)	O00Z N01D N00L 116.5(4)
N00J N005 C014 116.0(3)	N00G N00R C00U 115.5(3)	N00R C01F H01J 109.5
N00J N005 C01J 118.1(3)	N00G N00R C01F 118.7(3)	N00R C01F H01K 109.5
C014 N005 C01J 121.9(3)	C00U N00R C01F 123.2(3)	N00R C01F H01L 109.5
O00W N006 N004 116.4(4)	C00U N00R C01F 123.2(3)	H01J C01F H01K 109.5
O00W N006 O01E 128.5(6)	C00P C00S C00Q 117.8(3)	H01J C01F H01L 109.5
O01E N006 N004 115.0(6)	C00Q C00S N004 123.0(3)	H01K C01F H01L 109.5
O00X N007 C00T 118.7(4)	C00F C00T N007 118.3(3)	C00U C01G N00D 118.4(3)

O00X N007 O01A 125.1(4)	C00U C00T N007 119.2(3)	C00U C01G C010 122.8(3)
O01A N007 C00T 116.2(4)	C00U C00T C00F 122.5(3)	C010 C01G N00D 118.7(3)
O00N N009 N019 116.3(5)	C00T C00U N00R 120.3(3)	C00Q C01H N00H 122.1(3)
O013 N009 O00N 127.2(5)	C00T C00U C01G 117.6(3)	C018 C01H N00H 119.7(3)
O013 N009 N019 116.4(4)	C01G C00U N00R 122.0(3)	C018 C01H C00Q 118.0(3)
O01B N00D C01G 115.2(4)	C01C C010 N019 122.4(3)	N005 C01J H01D 109.5
O01I N00D O01B 125.0(5)	C01C C010 C01G 117.0(3)	N005 C01J H01E 109.5
O01I N00D C01G 119.9(5)	C01G C010 N019 120.5(3)	N005 C01J H01F 109.5
C00T C00F N00L 120.0(3)	O00Y N011 C00P 116.5(4)	H01D C01J H01E 109.5
C01C C00F N00L 122.8(3)	O01K N011 C00P 117.9(4)	H01D C01J H01F 109.5
C01C C00F C00T 117.2(3)	O01K N011 O00Y 125.6(4)	H01E C01J H01F 109.5
O00E N00G N00R 118.0(4)	O015 N012 C018 118.0(4)	N00L C01M H01M 109.5
O00E N00G O017 125.2(4)	O015 N012 O01L 125.0(5)	N00L C01M H01N 109.5
O00E N00G O017 125.2(4)	O01L N012 C018 116.9(4)	N00L C01M H01O 109.5
N001 N00H C016 119.7(3)	C00P C014 N005 123.4(3)	H01M C01M H01N 109.5
N001 N00H C01H 116.2(3)	C018 C014 N005 119.7(3)	H01M C01M H01O 109.5
C01H N00H C016 121.9(3)	C018 C014 C00P 116.9(3)	H01N C01M H01O 109.5
O003 N00J N005 116.8(3)	N00H C016 H01A 109.5	N004 C01N H01G 109.5
O00I N00J O003 125.8(4)	N00H C016 H01B 109.5	N004 C01N H01H 109.5
O00I N00J N005 117.3(4)	N00H C016 H01C 109.5	N004 C01N H01I 109.5
O00C N00K C01C 117.3(4)	H01A C016 H01B 109.5	H01G C01N H01H 109.5
O00V N00K O00C 126.0(4)	H01A C016 H01C 109.5	H01G C01N H01I 109.5
O00V N00K C01C 116.7(4)	H01B C016 H01C 109.5	H01H C01N H01I 109.5
C00F N00L C01M 120.9(4)	C014 C018 N012 119.1(3)	N019 C01O H01P 109.5
N01D N00L C00F 115.4(3)	C01H C018 N012 118.2(3)	N019 C01O H01Q 109.5
N01D N00L C01M 120.4(3)	C01H C018 C014 122.7(3)	N019 C01O H01R 109.5
O00A N00M O00O 126.0(4)	N009 N019 C010 115.8(4)	H01P C01O H01Q 109.5
O00A N00M C00Q 117.3(4)	N009 N019 C01O 118.9(4)	H01P C01O H01R 109.5
O00O N00M C00Q 116.7(4)	C010 N019 C01O 121.6(4)	H01Q C01O H01R 109.5

5.3 Hydrogen-bond analysis

Hydrogen-bond analysis. Intramolecular hydrogen-bond geometries were examined using Mercury based on the experimental crystallographic information files (CIFs) of compounds **1**-**3**. Contacts between O–H or N–H donors and nitro oxygen acceptors within the same molecule

were assessed by examining the H···O and donor···acceptor distances together with the D–H···O angles. Measurements were made using the atomic coordinates provided in the CIFs, without additional normalization of the X–H bond lengths. The atom labels and geometrical parameters are listed in Table S4-S6.

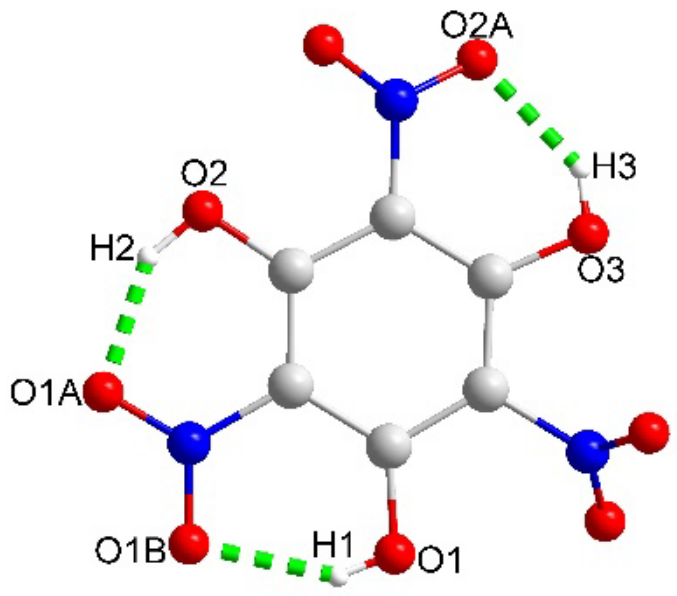

Figure S12. Crystal structure of **1** (Intramolecular hydrogen bonds).[1]

Table S4. Intramolecular hydrogen bonds in **1**.

D–H···A	D–H/Å	H···A/Å	D···A/Å	D–H···A/°
O(1)–H(1)···O(1B)	0.7521(229)	1.8688(244)	2.5264(21)	145.787(2820)
O(2)–H(2)···O(1A)	0.8783(370)	1.7481(474)	2.5351(32)	147.818(3815)
O(3)–H(3)···O(2A)	0.8231(391)	1.8636(337)	2.5733(25)	143.660(3142)

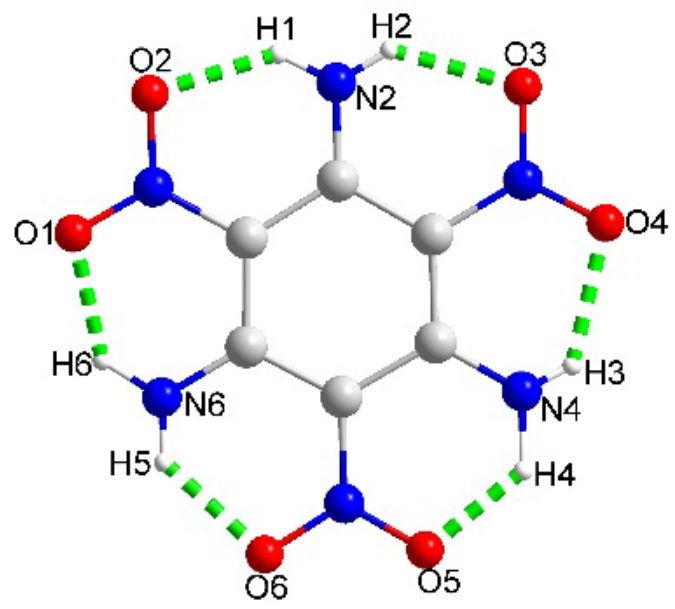

Figure S13. Crystal structure of **2** (Intramolecular hydrogen bonds).[2]

Table S5. Intramolecular hydrogen bonds in **2**.

D–H···A	D–H/Å	H···A/Å	D···A/Å	D–H···A/°
N(2)–H(1)···O(2)	0.8648	1.7974	2.4927	135.977
N(2)–H(2)···O(3)	0.8489	1.8383	2.4841	131.567
N(4)–H(3)···O(4)	0.7567	1.9479	2.5068	130.565
N(4)–H(4)···O(5)	1.0540	1.6915	2.4800	127.655
N(6)–H(5)···O(6)	0.8685	1.8162	2.5020	134.393
N(6)–H(6)···O(1)	0.9542	1.7704	2.4922	129.878

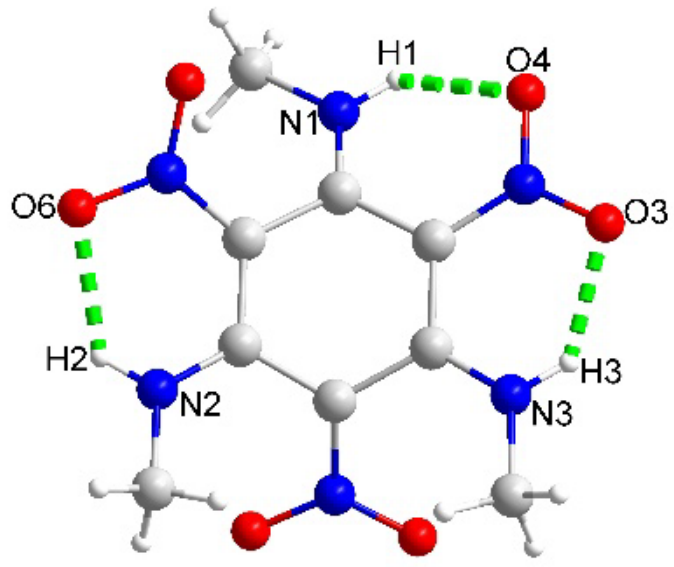

Figure S14. Crystal structure of **3** (Intramolecular hydrogen bonds).

Table S6. Intramolecular hydrogen bonds in **3**.

D–H···A	D–H/Å	H···A/Å	D···A/Å	D–H···A/°
N(1)–H(1)···O(4)	0.8600(28)	1.9493(32)	2.5930(46)	130.662(273)
N(2)–H(2)···O(6)	0.8597(29)	1.9546(37)	2.5817(53)	128.789(216)
N(3)–H(3)···O(3)	0.8603(24)	1.9049(36)	2.5726(47)	133.342(246)

6. Computational details

6.1 Heat of formation

The gas phase enthalpies of formation were calculated based on isodesmic reactions (Scheme S1, S2). The enthalpy of reaction is obtained by combining the MP2/6–311++G** energy difference for the reactions, the scaled zero point energies (ZPE), values of thermal correction (H_{corr}), and other thermal factors. The heats of formation of other compounds in Table S7 were obtained from the NIST WebBook. The solid state heats of formation were calculated with Trouton's rule according to the following equation (T represents either the melting point or the decomposition temperature when no melting occurs prior to decomposition). The calculated gas-phase heats of formation are listed in Table S7. The solid-phase heats of formation were subsequently obtained by subtracting the corresponding sublimation enthalpies

$$\Delta H_{sub}=188/Jmol^{-1}K^{-1}\bullet T$$

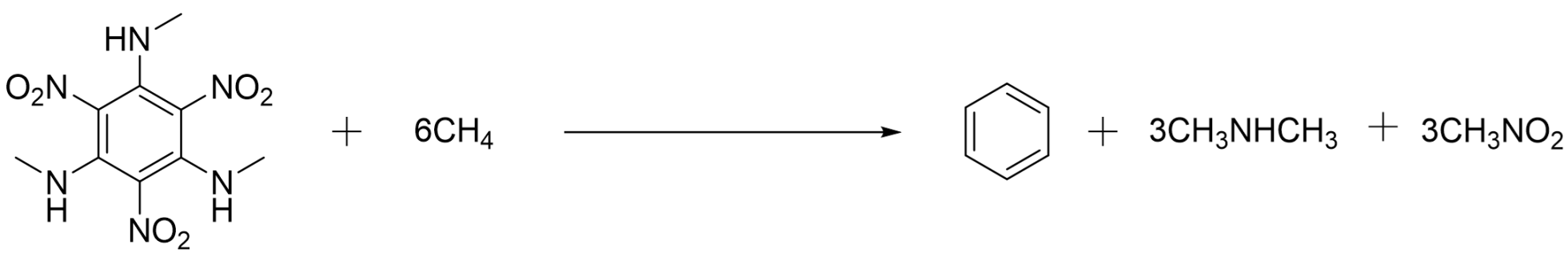

Scheme S1. Isodesmic reactions for calculating heats of formation for **3**, respectively.

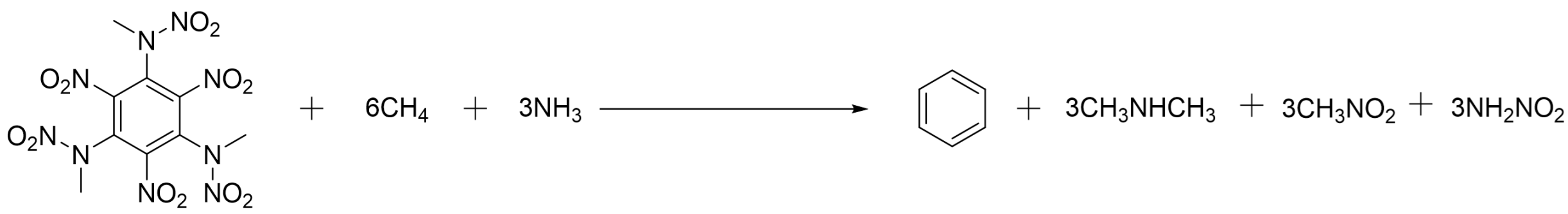

Scheme S2. Isodesmic reactions for calculating heats of formation for **5**, respectively.

Table S7. Calculated Total Energy (E_0), Zero Point Energy (ZPE), values of Thermal Correction (H_{corr}) and calculated gas-phase heat of formation of the compounds.

Compound	ZPE/a.u.	H_{corr}/a.u.	E_0/a.u.	ΔH_f(gas) (kJ/mol)
**3**	0.244144	0.264249	-1127.132404	73.2
**5**	0.246423	0.276096	-1739.376977	158.3
CH_4	0.044793	0.048605	-40.3796224	-74.6
Benzene	0.100451	0.105797	-231.5842377	82.9
CH_3NHCH_3	0.092338	0.097686	-134.7787219	-23.8
CH_3NO_2	0.049856	0.055129	-244.4784875	-74.3
NH_3	0.034372	0.03819	-56.4154644	-45.9
NH_2NO_2	0.039264	0.043917	-260.4931874	-3.3

6.2 Computational details for IRI analysis

Computational details for IRI analysis. Single-point calculations were performed at the B3LYP/6-311++G(d,p) level using Gaussian 16 A on molecular geometries extracted from the experimental crystal structures of compounds **1**–**5**. No geometry optimization was performed prior to the IRI analysis. The resulting electron densities were analyzed using Multiwfn [version 2026.1.12] to generate interaction region indicator (IRI) maps following the method developed by Lu and Chen.[3-5]

6.3 LOL-π analysis

The π-resolved localized orbital locator (LOL-π) was evaluated for compounds **1**–**5** using Multiwfn (version 2026.1.12) from wavefunctions obtained with Gaussian at the B3LYP/6-311++G(d,p) level.[6] The analysis considered the selected occupied π orbitals. All LOL-π isosurfaces were visualized at a common dimensionless isovalue of 0.5. Approximately face-on and side views are provided in Figure S15 to show the spatial distributions around the benzene rings.

At this isovalue, compounds **1** and **3** display local interruptions in the ring-associated isosurfaces, whereas compounds **4** and **5** show more continuous distributions around the ring. These differences provide a qualitative description of the π-electron localization and delocalization patterns. The size and continuity of the displayed isosurfaces do not directly measure π-electron populations or aromatic stabilization energies and depend on the selected isovalue.

Compound **1** remains nearly planar despite its interrupted isosurface, indicating that this feature alone is insufficient to explain the ring puckering of compound **3**. Moreover, substituent identity and molecular geometry both vary across the series. The LOL-π results therefore complement the structural and interaction analyses but do not isolate an electronic driving force for puckering.

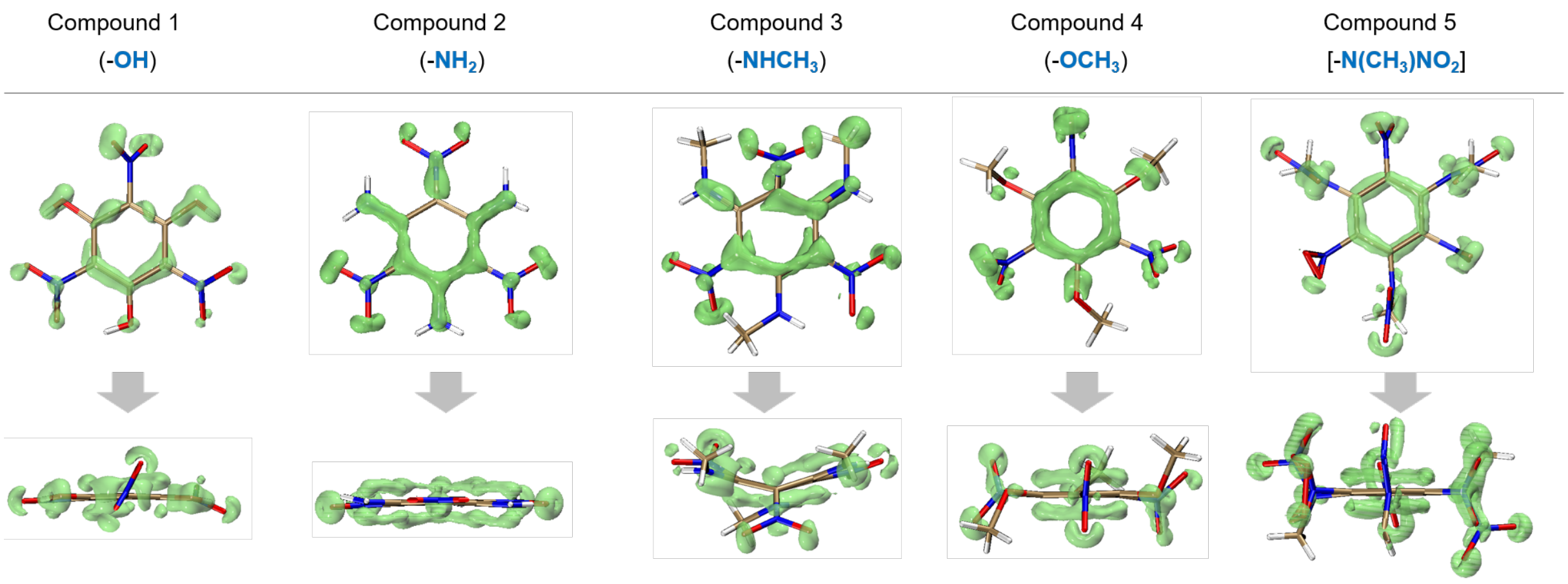

Figure S15. LOL-π isosurfaces of compounds **1**–**5**.

7. Thermal stability

Differential scanning calorimetry (DSC/TG) showed that the decomposition temperature of **3** is 275.98 °C.

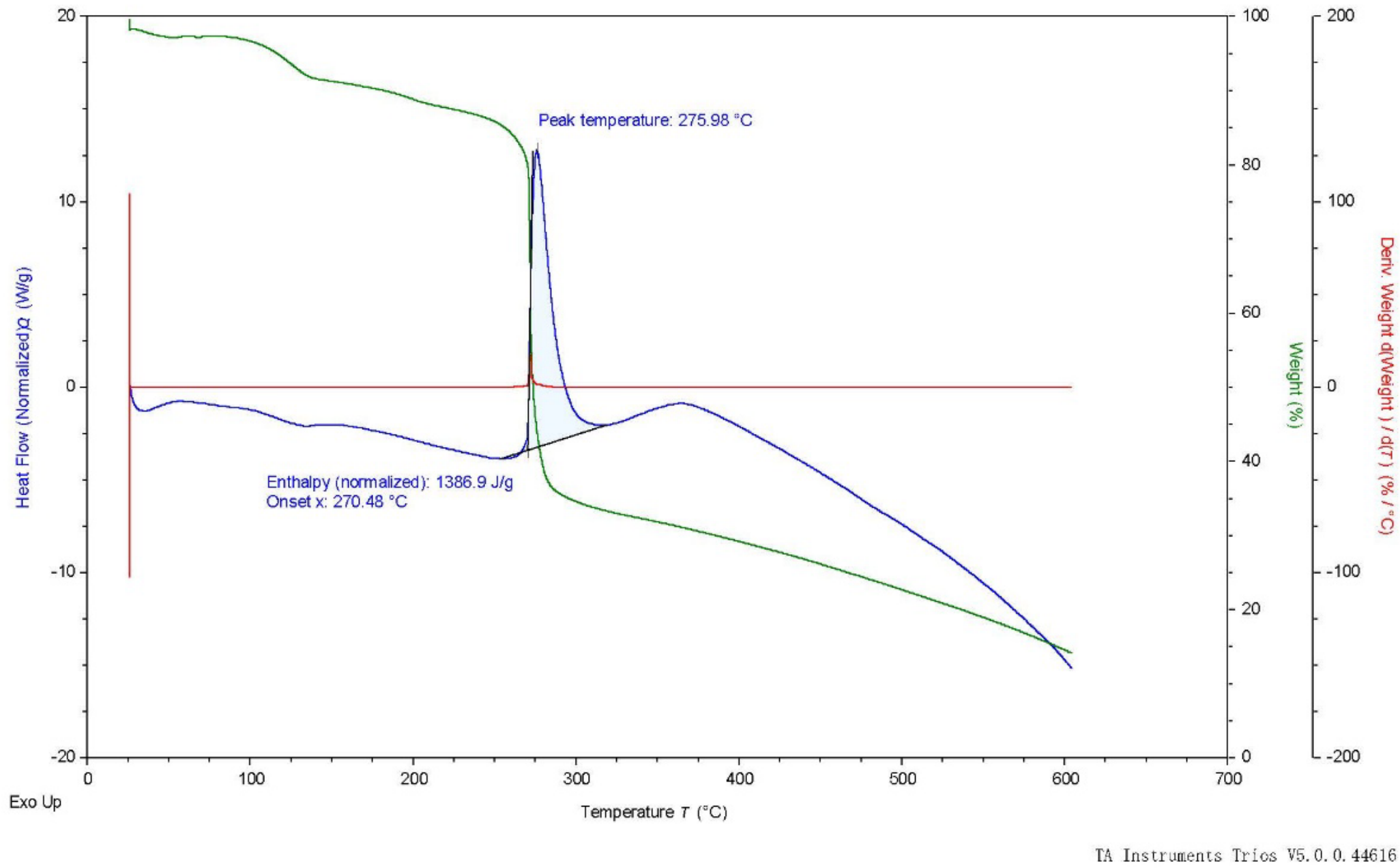

Figure S16. DSC plot of **3**.

Differential scanning calorimetry (DSC/TG) showed that the decomposition temperature of **5** is 202.1 °C.

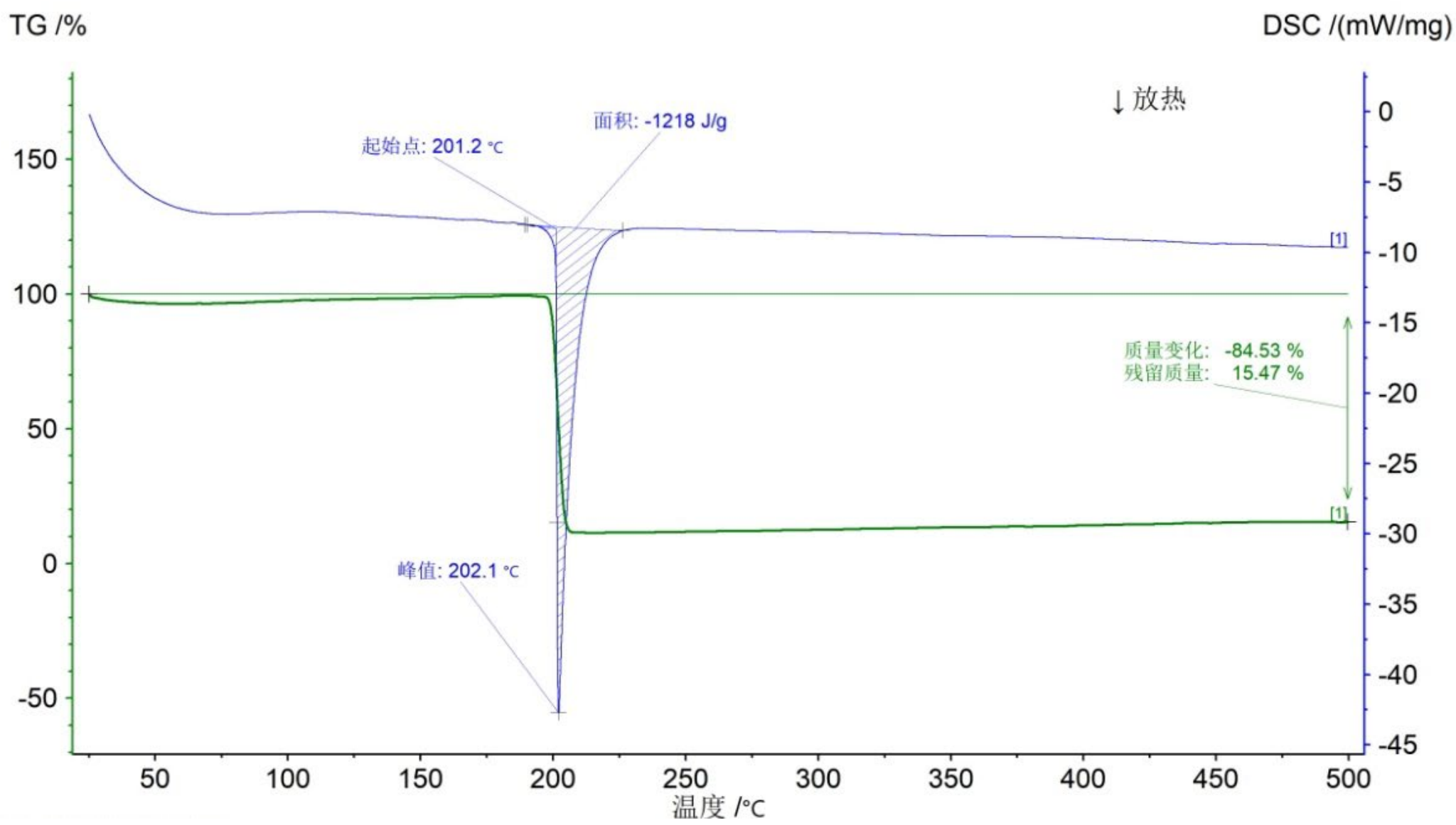

Figure S17. DSC plot of **5**.

8. Energetic properties

Table S8. Physiochemical properties and energetic parameters of energetic benzenes.

Comp.	d[a]	ΔH_f[b]	D[c]	P[d]	T_d[e]	IS[f]	FS[g]
**3**	1.611	-30.0	6847	17.1	276	13	168
**5**	1.692	69.2	7838	25.7	200	9	96
TNT[h]	1.650	-67.0	6881	19.5	295	15	353
TNTNB[i]	1.964	392.6	9510	42.2	65	1	5

[a] Density measured by a gas pycnometer at 296K (g/cm^3); [b] Calculated solid-phase heats of formation for compounds 3 and 5, kJ/mol; [c] Detonation velocity (calculated with EXPLO5 v6.05), m/s; [d] Detonation pressure (calculated with EXPLO5 v6.05), GPa; [e] Decomposition temperature under N_2, °C; [f] Impact sensitivity, J; [g] Friction sensitivity, N; [h] Ref. [7] ; [i] Ref. [8].

9. variable-temperature FTIR and UV-vis measurements

Variable-temperature Fourier-transform infrared (VT-FTIR) measurements were performed on a Bruker VERTEX 80v spectrometer (Germany) under nitrogen atmosphere. The temperature was raised from 30 °C to 150 °C with a heating rate of 5 °C/min, and spectra were recorded at every 10 °C once the temperature reached stabilization. Variable-temperature UV-Vis absorption spectra were acquired on a Perkin Elmer Lambda 750(s) spectrophotometer (USA) in the wavelength range of 200–800 nm. The temperature was adjusted from 30 °C to 150 °C, and data were collected at every 20 °C after thermal equilibration. The VT-FTIR spectra of compound 3 exhibit non-monotonic changes in band shape and relative intensity in the approximately 3300–3000 cm^{-1} region, where hydrogen-bonded N–H stretching vibrations are expected to contribute. These changes may reflect perturbations of the local hydrogen-bonding environment, although overlapping bands and temperature-dependent intensity variations limit a more specific interpretation. The corresponding UV–vis spectra largely retain their overall profile over 30–150 °C. These measurements do not directly determine whether the benzene ring conformation changes upon heating.

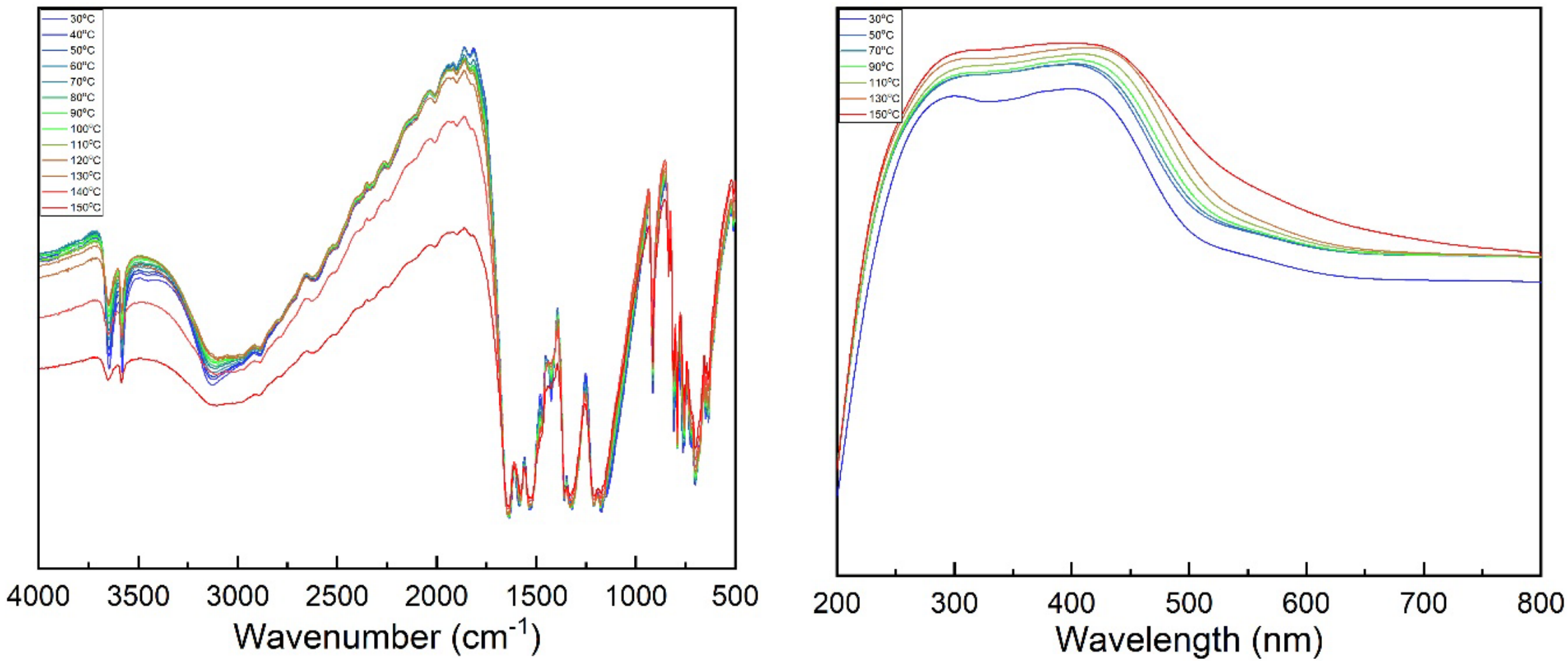

Figure S18. VT-FTIR and UV-Vis of **1**.

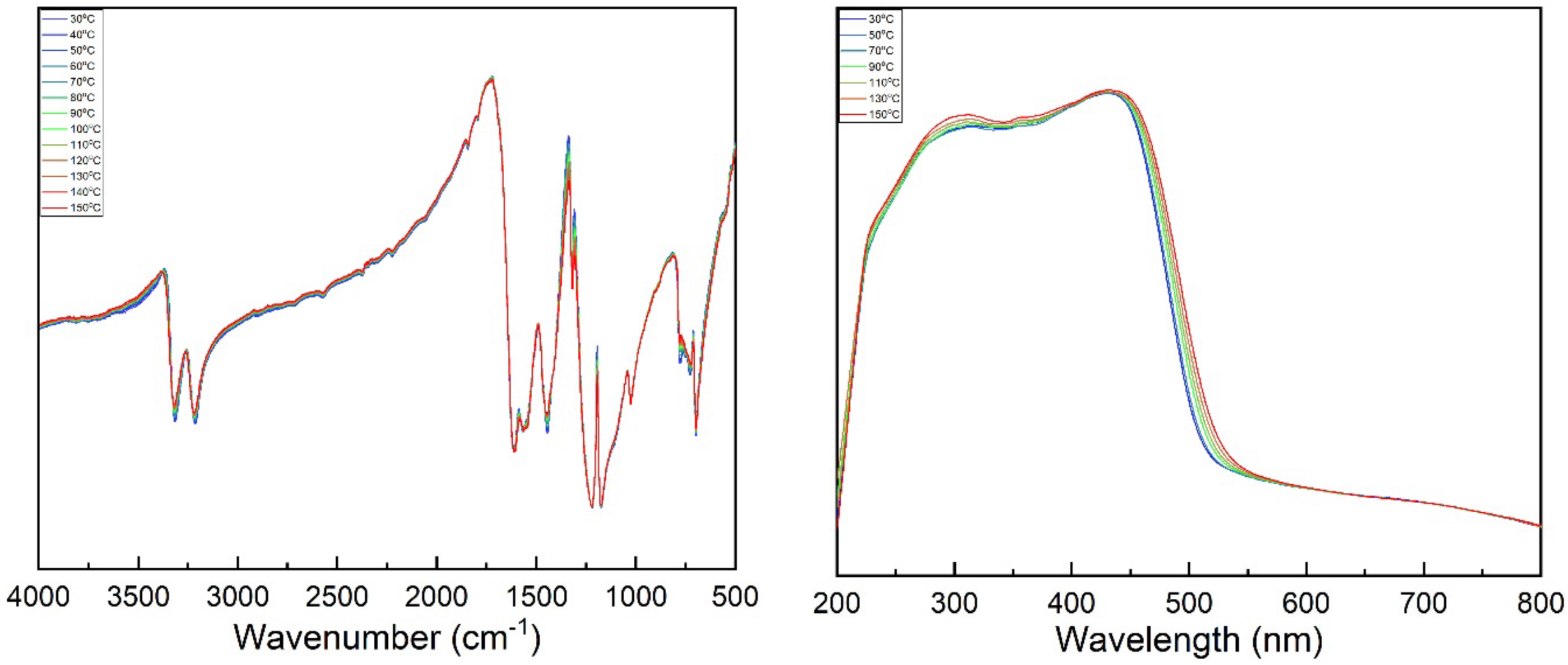

Figure S19. VT-FTIR and UV-Vis of **2**.

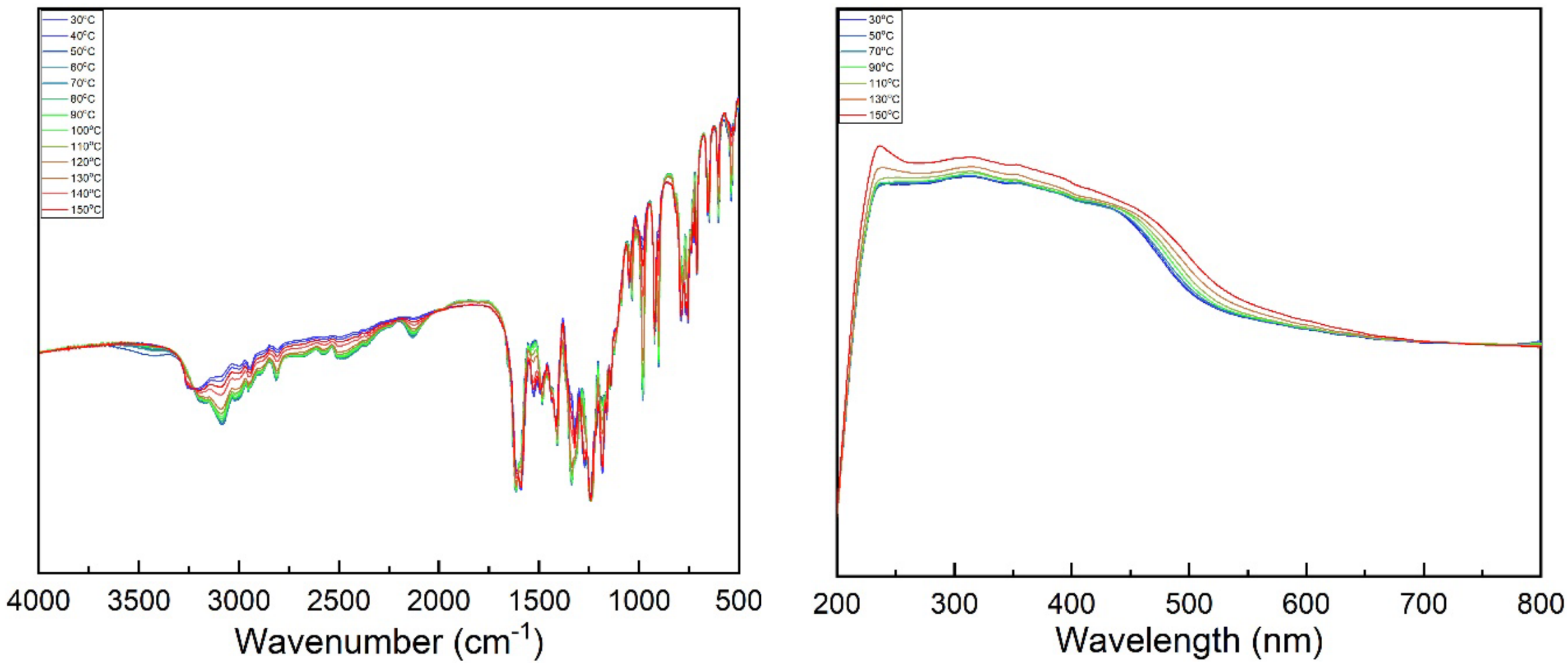

Figure S20. VT-FTIR and UV-Vis of **3**.

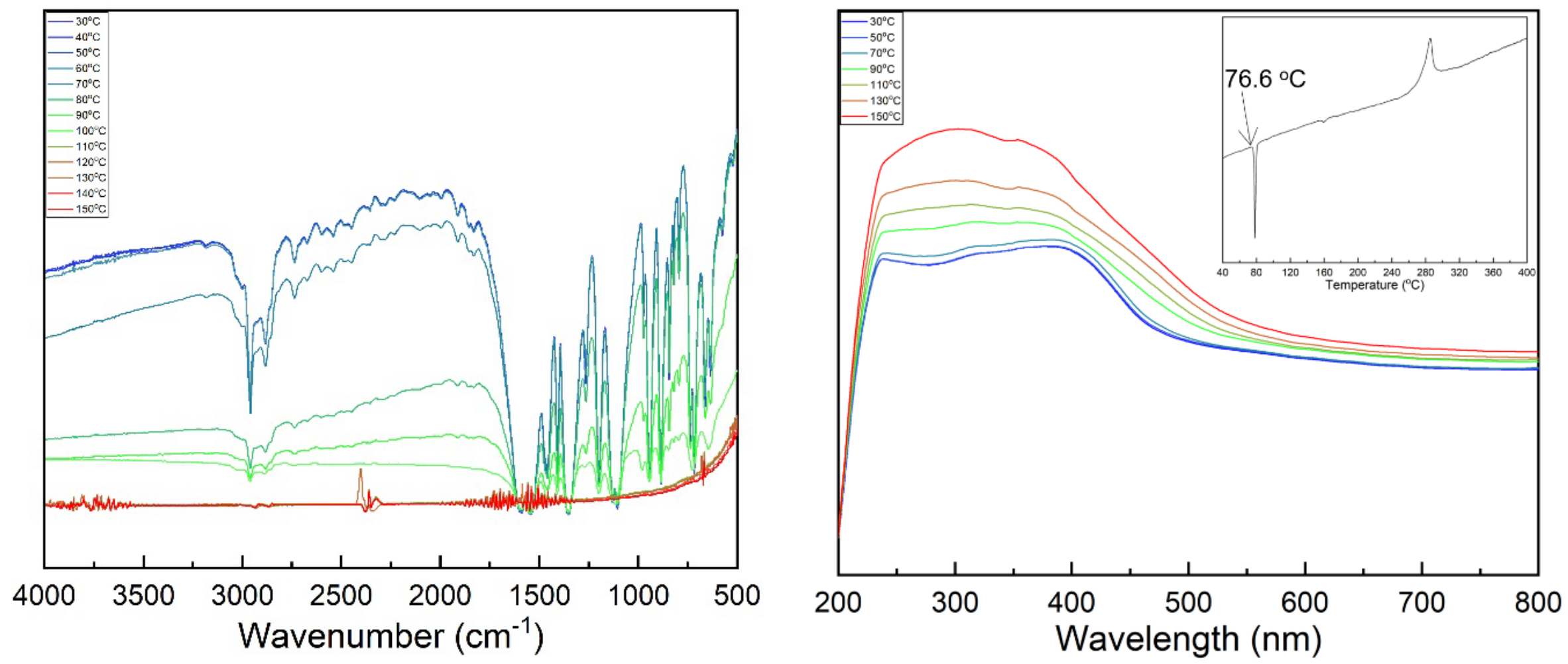

Figure S21. VT-FTIR and UV-Vis of **4** (The melting point of **4** is 76.6 °C).

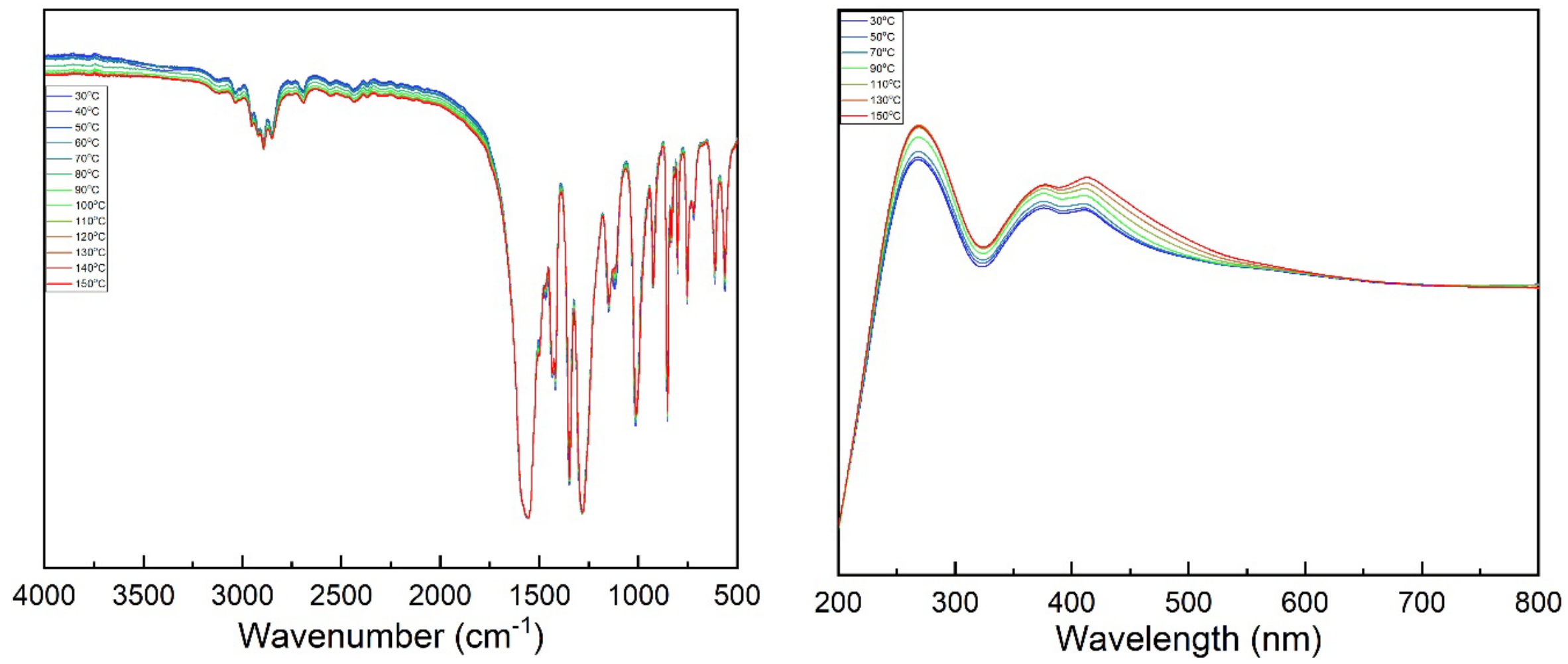

Figure S22. VT-FTIR and UV-Vis of **5**.

REFERENCE

1. The crystal data of **1** was cited from the Cambridge Crystallographic Data Centre with CCDC number of 128229.

2. The crystal data of **2** (TATB) was cited from the Cambridge Crystallographic Data Centre with CCDC number of 1266837.

3. T. Lu and F. Chen., "Multiwfn: A multifunctional wavefunction analyzer," *Journal of Computational Chemistry* 33, (2012): 580-592.

4. T. Lu., "A comprehensive electron wavefunction analysis toolbox for chemists, Multiwfn," *The Journal of Chemical Physics* 161, (2024): 082503.

5. T. Lu and Q. Chen., "Interaction Region Indicator: A Simple Real Space Function Clearly Revealing Both Chemical Bonds and Weak Interactions," *Chemistry–Methods* 1, (2021): 231-239.

6. T. Lu and Q. Chen., "A simple method of identifying π orbitals for non-planar systems and a protocol of studying π electronic structure," *Theoretical Chemistry Accounts* 139, (2020): 25.

7. M. Li, ZW. Zeng, YQ. Dong, J. Liu, YJ. Liu, W. Huang, GB. Cheng, C. Xiao, YX. Tang "[5,5]-Fused Heterocycle-Bridged Bistetrazoles Enable High-Energy and Insensitive Nitrogen-Rich Materials". Small, (2026): e73287.

8. Q. Sun, N. Ding, CF. Zhao, Q. Zhang, SW. Zhang, S. Li, SP. Pang "Full-nitro-nitroamino cooperative action: Climbing the energy peak of benzenes with enhanced chemical stability". *Science Advances* 8, (2022): eabn3176.
